# Role of increased IGFBP2 in trophoblast cell proliferation and recurrent spontaneous abortion development: A pilot study

**DOI:** 10.14814/phy2.15939

**Published:** 2024-02-05

**Authors:** Li Ji, Ziying Jiao, Lin Zhang, Jia Shi, Qianqian Wan, Chunzhi Qian, Han Wang, Xiaoyan Cao, Bing Shen, Lijuan Jiang

**Affiliations:** ^1^ The First Clinical Medical College Nanjing University of Traditional Chinese Medicine Nanjing China; ^2^ Department of Obstetrics and Gynecology Lu'an Traditional Chinese Hospital, The Affiliated Hospital of Anhui University of Chinese Medicine Lu'an China; ^3^ Dr. Neher's Biophysics Laboratory for Innovative Drug Discovery, State Key Laboratory of Quality Research in Chinese Medicine Macau University of Science and Technology Macau China; ^4^ Department of Gynecology The First Affiliated Hospital of Yunnan University of Traditional Chinese Medicine Kunming China; ^5^ School of Basic Medicine Sciences Anhui Medical University Hefei China

**Keywords:** IGFBP2, proliferation, proteomics, recurrent spontaneous abortion, RNA‐seq, trophoblast

## Abstract

Recurrent spontaneous abortion (RSA) is a serious condition that adversely affects women's health. Differentially expressed proteins (DEPs) in plasma of patients experiencing RSA is helpful to find new therapeutic targets and identified with mass spectrometry. In 57 DEPs, 21 were upregulated and 36 were downregulated in RSA. Gene ontology analyses indicated that identified DEPs were associated with cell proliferation, including significantly downregulated insulin‐like growth factor binding protein 2 (IGFBP2). Immunohistochemical result using clinical decidual tissues also showed that IGFBP2 expression was significantly decreased in RSA trophoblasts. Cell proliferation assay indicated that IGFBP2 treatment increased the proliferation and mRNA expressions of PCNA and Ki67 in trophoblast cells. Transcriptome sequencing experiments and Kyoto Encyclopedia of Genes and Genomes analyses revealed that gene expression for components in PI3K‐Akt pathway in trophoblasts was significantly upregulated following IGFBP2 treatment. To confirm bioinformatics findings, we did cell‐based experiments and found that treatment of inhibitors for insulin‐like growth factor (IGF)‐1 receptor‐PI3K‐Akt pathway significantly reduced IGFBP2‐induced trophoblast cell proliferation and mRNA expressions of PCNA and Ki67. Our findings suggest that IGFBP2 may increase trophoblast proliferation through the PI3K‐Akt signaling pathway to affect pregnancy outcomes and that IGFBP2 may be a new target for future research and treatment of RSA.

## 1 INTRODUCTION

One percent of couples trying to get pregnant have recurrent spontaneous abortion (RSA), which is defined as the loss of three or more consecutive pregnancies (Rai & Regan, 2006). Repeated miscarriages have a huge impact not only on the psychological well‐being of individuals and their families but also on national health care systems and economic development. Recently, many reasons have been reported to induce spontaneous abortion, for example, fetal chromosomal abnormalities, alcohol consumption, smoking, cocaine use, fatigue, the number of pregnancies, age at primary birth and age at the last pregnancy, and some diseases including diabetes, celiac disease, autoimmune conditions, cervicitis, vaginitis, uterine congenital anomalies, uterine leiomyoma, intrauterine adhesions, HIV infection, hepatitis B virus infection, merkel cell polyomavirus infection, syphilis, and malaria (Alves & Rapp, 2023; Griebel et al., 2005; Hu et al., 2018; Khalid et al., 2022; Mazziotta et al., 2021). In addition, environmental risk factors such as arsenic, lead, and organic solvents exposure are associated to spontaneous abortion (Alves & Rapp, 2023; Griebel et al., 2005). Besides, spontaneous abortion may increase the risk of premature mortality, especially leading to death from cardiovascular disease (Wang et al., 2021). For the purpose of early detection and treatment, it is crucial to identify possible biomarkers and therapeutic targets linked to RSA. Several studies show that the defects of trophoblast cells may cause RSA (Chen et al., 2023; Luo et al., 2023; Ma et al., 2023). Therefore, trophoblast cell function is closely associated with RSA occurrence and development. Even though it may have a number of causes, the underlying molecular processes behind RSA have not been fully clarified (Larsen et al., 2013).

In numerous mammalian species, the role of insulin‐like growth factor, also known as IGF, and IGF binding proteins (IGFBPs) in follicular differentiation during the development of ovaries has been extensively characterized (Mazerbourg et al., 2003). In all of these species, IGFs and a number of small‐molecular‐weight IGFBPs, particularly IGFBP‐2 and IGFBP‐4, are thought to stimulate or inhibit follicular development and maturation (Mazerbourg & Monget, 2018). In ovarian cancer, IGFBPs can serve as predictable biomarkers and potential therapeutic targets (Prayudi et al., 2020; Tan et al., 2023; Zheng et al., 2020). IGFBP‐2 is primarily expressed in fetal tissues that are highly proliferative (Li et al., 2020) including the early postimplantation progenitor cells of the upper layer, the ventricular region of the rostral neuroepithelium (Pintar et al., 1991; Wood et al., 1992), the apical ectodermal crest (van Kleffens et al., 1998), and progenitor cells of the spleen (Pintar et al., 1991) and liver (Streck & Pintar, 1992). In rats, IGFBP‐2 expression has been shown to exist in the prenatal neocortex in a pattern consistent with that of neonatal astrocytic expression but is greatly reduced during postpartum development (Lee et al., 1993). In addition, serum IGFBP2 has a potential promising biomarker for detecting ovarian cancer (Prayudi et al., 2020). However, the role of IGFBP2 in trophoblast cell proliferation and RSA development is still unknown.

In the present study, in order to find differentially expressed proteins (DEPs) in plasma samples collected from pregnant women with versus without RSA, we first employed liquid chromatography with tandem mass spectrometry (LC‐MS/MS). The identified DEPs were assessed using bioinformatics analysis to determine which of them and their associated signal transduction pathways may play key roles in RSA. Then, we determined whether IGFBP‐2 as an important DEP is associated with the proliferation of trophoblast cells, which are involved in attaching the developing embryo to the uterus wall, protecting the embryo, and forming a part of the placenta.

## 2 MATERIALS AND METHODS

### 2.1 Materials

In December 2021, plasma samples were collected from eight women with ongoing pregnancy at the Lu'an Traditional Chinese Hospital: four from patients after they received a diagnosis of RSA but before any treatment was initiated, and from four healthy women with normal pregnancies, that is, no pregnancy disorder and undergoing no treatments at the time of sample collection (Table 1). In addition, we have also excluded the patients with some diseases, including cancer, systemic sclerosis, idiopathic pulmonary fibrosis and pulmonary arterial hypertension, maybe affecting the expression of serum IGFBP2 level (Dong et al., 2020; Guiot et al., 2016, 2021; Yang et al., 2020). The 2012 recurrent pregnancy loss recommendations from the American Society for Reproductive Medicine served as the foundation for the diagnostic criteria for RSA (Practice Committee of the American Society for Reproductive Medicine, 2012). All patients were examined by obstetrician‐gynecologists.

**TABLE 1:** Demographic characteristics of participants for LC‐MS/MS analysis.

Groups	Age (years)	Childbearing history	Previous number of spontaneous abortions
Health women	27	2‐0‐0‐2	0
	25	1‐0‐0‐1	0
	32	1‐0‐1‐1	0
	33	2‐0‐0‐2	0
Recurrent spontaneous abortion women	37	0‐0‐3‐0	3
	33	0‐0‐2‐0	2
	32	1‐0‐2‐1	2
	28	1‐0‐2‐1	2

*Note*: childbearing history is showing the number of full‐term birth‐premature delivery‐abortion‐surviving child.

All study procedures were approved by Lu'an Traditional Chinese Hospital Medical Ethics Committee (2021‐KY‐LL‐001), and all RSA and healthy women with normal pregnancies provided written informed consent.

### 2.2 Protein extraction, separation, and quantification

After first creating the protein solution based on a standard curve, we utilized the columns (NP0007, Thermo Fisher, Massachusetts, USA) to extract the proteins in the eight original plasma samples as per the manufacturer's instructions. The samples were separated by SDS‐PAGE using 10% gels. Final staining with Coomassie bright blue.

### 2.3 Enzymatic hydrolysis of proteins and high performance LC and LC‐MS/MS analysis

According to previous studies (Li et al., 2022; van Zundert et al., 2023), the quantified protein (60 μg) of each sample was added into a centrifuge tube, and then added dithiothreitol (5 μL of 1 M) the sample following incubation at 37°C for 1 h. Iodoacetamide (20 μL of 1 M) was added to the sample. After another incubation in the dark at room temperature for 1 h, the samples were centrifuged at 14,000 × *g* for 20 min at 4°C. The precipitate was collected, and 100 μL of UA buffer (8 M urea, 100 mM Tris‐HCl, pH 8.0) was added. After ultracentrifugation, the resulting pellet was collected, and this process was repeated for two times. NH_4_HCO_3_ (100 μL of 50 mM) was added and then centrifuged. The pellet was collected and this process was repeated for three times. Finally, the pellet was digested with trypsin according to the (mass) protein to enzyme ratio of 50:1 for 12–16 h at 37°C. Next, we used nanoliter flow rate and high‐performance liquid chromatography with a liquid phase system to separate the samples. Firstly, 95% of phase A was used to equilibrate the chromatographic column. An analytical column was used to separate the samples after they had been loaded by an autosampler onto an MS pre‐column. The mobile phases were Phases A and B, containing 0.1% formic acid in both water and acetonitrile. The LC‐elution gradient parameters were as follows: 0–10 min, phase A at 94% and phase B at 6%; 10–15 min, phase A decreased linearly from 94% to 90%, phase B increased linearly from 6% to 10%; 15–70 min, phase A decreased to 70%, phase B increased to 30%; 70–80 min, phase A decreased to 60%, phase B increased to 40%; 80–80.1 min, phase A decreased to 5%, phase B increased to 95%; 80.1–85 min, phase A remained at 5%, phase B remained at 95%; 85–85.1 min, phase A increased to 94%, phase B decreased to 6%; 85.1–88 min, phase A remained at 94%, phase B at 6%. At this time, all liquid‐phase flow rates were 600 nL/min. Each sample underwent capillary high‐performance LC sample separation and MS analysis using a Q Exactive HF‐X mass spectrometer (Thermo Scientific, USA).

### 2.4 Protein sequence database search and data analysis

According to an analysis flowchart in Figure S1, Proteome Discoverer software (version 2.4, Thermo Fisher Scientific, USA) was employed for data analysis. SEQUST search engine was used to identify peptide using human proteome databases that include sequences from UniProt (https://www.uniprot.org/). Decoys were generated with the revert function, and the options were using as following: Peptide mass tolerance: ±15 ppm, MS/MS tolerance: 0.02 Da, enzyme: trypsin, missed cleavage: 2, fixed modification: Carbamidomethyl (C), variable modification: oxidation (M), database pattern: decoy. We set to 0.01 for the false discovery rate (FDR) for peptides and proteins. Upregulated or downregulated proteins with a relative quantitative *p* value < 0.05 and a fold change (FC) of 1.5 between two groups were considered DEPs. Upregulated proteins had an FC ≥1.5 and a value of *p* < 0.05, whereas downregulated proteins had an FC ≤0.667 and *p* < 0.05. No difference had FCs between 0.667 and 1.5 or values of *p* > 0.05.

### 2.5 Immunohistochemistry

We obtained the decidual tissues of additional five healthy women with normal pregnancies and five RSA patients from the Department of Pathology of the Lu'an Traditional Chinese Hospital within the period of study approved by Lu'an Traditional Chinese Hospital Medical Ethics Committee (2021‐KY‐LL‐001) with the written informed consent of the objects. The information of all participates were shown in Table 2. The samples were fixed with 4% paraformaldehyde and then dehydrated and embedded. The tissue sections with 5‐μm‐thick were cut out, dewaxed, and washed by using graded alcohol solutions and tap water. Endogenous peroxidase enzyme activity was blocked, and antigen retrieval was performed in tissue sections. After then, the sections were washed with sterile phosphate‐buffered saline (PBS) and incubated with specific primary rabbit anti‐IGFBP2 antibody (1:100, #bs‐1108R, Beijing Biosynthesis Biotechnology CO., LTD., Beijing, China) overnight. In next day, the sections were washed with PBS again and incubated with horseradish peroxidase‐conjugated secondary antibody (#PV‐6000, 1:10000, Beijing Zhong Shan‐Golden Bridge Biological Technology Co., Ltd., Beijing, China). Then the sections were treated with diaminobenzidine (#ZLI‐9018, Beijing Zhong Shan‐golden Bridge Biological Technology Co., Ltd., Beijing, China) for 1–3 min and counterstained with hematoxylin (#BA‐4041, BaSO Biological Technology Co., Ltd., Zhuhai, China). Eventually, the image data were photographed using a light microscopy (OLYMPUS, CX41 with 20× objective lens and software: Image‐Pro Plus. 7.0).

**TABLE 2:** Demographic characteristics of participants for immunohistochemistry assay.

Groups	Age (years)	Childbearing history	Previous number of spontaneous abortions
Health women	29	2‐0‐1‐2	0
	30	1‐0–0‐1	0
	30	1‐0‐1‐1	0
	38	2‐0‐1‐2	0
	26	1‐0‐2‐1	1
Recurrent spontaneous abortion women	32	0‐0‐3‐0	2
	24	0‐0‐2‐0	2
	25	1‐0‐3‐1	3
	41	1‐0‐2‐1	2
	23	0‐0‐2‐0	2

*Note*: childbearing history is showing the number of full‐term birth‐premature delivery‐abortion‐surviving child.

### 2.6 Cell culture and proliferation assay

Human chorionic trophoblast cells (HTR‐8/SVneo) were obtained from ATCC (American Type Culture Collection, Manassas, VA, USA) and cultured in Roswell Park Memorial Institute (RPMI)‐1640 media (BasalMedia, Shanghai, China) with 10% fetal bovine serum (#A5669401, Thermo Fisher Scientific, USA) and 1% penicillin–streptomycin at 37°C with saturated humidity, 5% CO_2_. Cell proliferation assay was performed as a previous study (Mu et al., 2023). Briefly, cells at the logarithmic growth phase were collected for experiments. The proliferative ability of HTR‐8/SVneo cells was assessed using a Cell Counting Kit‐8 (CCK8, #C0037, Beyotime, China) assay in accordance with the manufacturer's instructions. Using a microplate scanner (BioTek Synergy 2, USA), the absorbance of each sample well was determined at a wavelength of 450 nm. The cells were treated with IGFBP2 (#HY‐P7368, MedChemexpress CO., LTD., USA) alone or combined with 0.2 μM picropodophyllin (PPP, #407247, Merk SA, Darmstadt, Germany), 0.1 μM ZSTK474 (#SML3194, Merk SA, Darmstadt, Germany), or 0.2 μM afuresertib (#A413769, Aladdin Biochemical Technology CO., LTD., Shanghai, China).

### 2.7 RNA sequencing

RNA sequencing was performed according our previous study (Huang et al., 2020). Briefly, HTR‐8/SVneo cell samples were incubated with Trizol reagent (15596‐026, Invitrogen, USA) at room temperature for 5 min. A spectrophotometer (Thermo Fischer Scientific, MA, USA) was used to determine the concentration and purity (absorption ratio of 260–230 nm) of the total RNA to ensure a 260/230 absorption ratio of 2.0. We then used a VAHTS Universal V8 RNA‐seq Library Prep Kit for Illumina (#NR605‐01/02, Vazyme Biotech Co., Ltd, China) to establish the library and a high‐throughput Illumina HiSeq 2500 sequencing platform to sequence the RNA samples to a length of 150 base pairs. According to an analysis flowchart in Figure S1, Trimmomatic was used to trim the RNA‐Seq FastQ raw data to remove the adapter sequence and reduce the quality of the readings (Bolger et al., 2014). The clean data's quality was assessed using FastQC software (Hwang et al., 2018). Thereafter, transcript expression levels were determined (fragments per kilobase of transcript per million mapped fragments, FPKM) (Yu et al., 2014). Differences in transcription expression between samples were determined. Kallisto software and the edgeR package (http://bioconductor.org/packages/2.4/bioc/html/edgeR.html) were used to examine the abundances of known mRNA transcripts as well as the differences in the number of reads (Bray et al., 2016; Robinson et al., 2010; Yang et al., 2018). Bioinformatics analyses were performed on the transcriptomes that were differentially expressed, defined at a threshold value of a 2‐fold change (Park et al., 2010).

### 2.8 Gene ontology (GO), Kyoto encyclopedia of genes and genomes (KEGG) enrichment analysis

We performed functional and pathway enrichment analyses using Metascape, a web‐based resource for gene annotation, visualization, and integration discovery (http://metascape.org), according our previous study (Fang et al., 2019). As a biological database, GO database is used for storing, organizing, and disseminating annotation information about genes and proteins (Ashburner et al., 2000). The ontologies for DEPs and differentially expressed genes (DEGs) were obtained by using Metascape.

As a comprehensive bioinformatics database, KEGG provides rich gene and protein annotation data along with associated information on signaling pathways (Kanehisa & Goto, 2000). KEGG pathway analysis and visualization for DEGs were conducted using the KOBAS online analysis database (http://kobas.cbi.pku.edu.cn/). A two‐sided *p* value less than 0.05 was considered statistically significant (Xie et al., 2011).

### 2.9 Reverse transcription‐quantitative (RT‐qPCR)

qPCR experiment was used to examine mRNA expression level. Briefly, a cDNA Reverse Transcription Kit (#11141ES60, Yeasen Biotechnology Co., Ltd., China) was used to synthesize cDNA by adding total RNA. A Roche LightCycler 480 II PCR equipment (Roche, Switzerland) and a kit of TB Green Premix Ex TaqTM II (#RR820A TaKaRa Bio, Japan) were used for qPCR reaction. Thermocycler steps were 5 min at 95°C, followed by 40 cycles of 15 s at 95°C and 30 s at 61°C. The primers used for qPCR were as follows: forward primer for β‐actin (*ACTB*): 5′‐ATCCACGAAACTACCTTCAACTCCAT‐3′, reverse primer for *ACTB*: 5′‐CATACTCCTGCTTGCTGATCCACATC‐3′; forward primer for proliferating cell nuclear antigen (*PCNA*): 5′‐ACACTAAGGGCCGAAGATAACG −3′, reverse primer for *PCNA*: 5′‐ACAGCATCTCCAATATGGCTGA‐3′; forward primer for marker of proliferation Ki‐67 (*MKI67*): 5′‐ AGAAGAAGTGGTGCTTCGGAA‐3′, reverse primer for *MKI67*: 5′‐ AGTTTGCGTGGCCTGTACTAA‐3′. The primers for qPCR were obtained from Sangon Biotech Co. Ltd., China, with a polyacrylamide gel electrophoresis purification. The expression levels of the mRNA were calculated by a $2^{-\Delta \Delta C_{t}}$ method relative to the endogenous control genes: β‐actin/18S rRNA.

### 2.10 Statistical analysis

A statistical software (GraphPad Prism 7.0) was used to analyze the data using two‐tailed Mann–Whitney and Fisher exact tests. To compare data in ≥3 groups, one‐way analysis of variance (ANOVA) was conducted. The values are expressed as mean ± SD. Values of *p* < 0.05 were considered statistically significant.

## 3 RESULTS

### 3.1 SDS‐page

Using SDS‐PAGE, we isolated protein in plasma samples from four patients with RSA and from four healthy pregnant controls. All proteins in the molecular range of 11–245 kDa were isolated, and no proteins were degraded, indicating that the samples contained sufficient proteins for use in subsequent experiments (Figure S2).

### 3.2 LC‐MS/MS analysis

**FIGURE 1:**
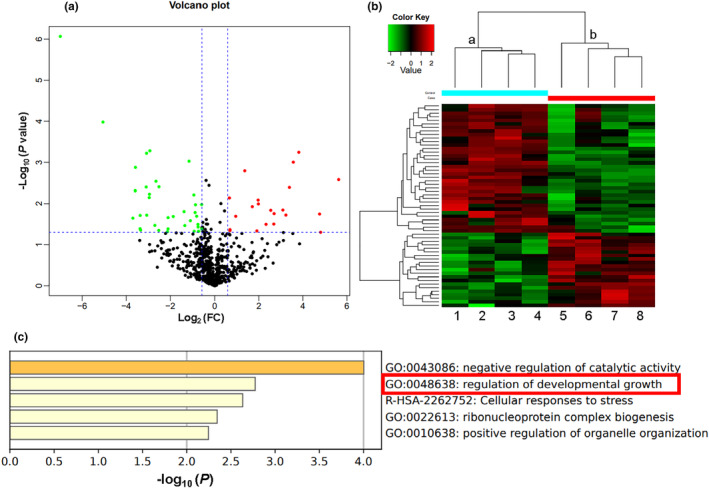
Volcano plot and heat maps of the differentially expressed proteins. (a) Volcano plot of proteins with significantly changed levels of expression in patients with recurrent spontaneous abortion (RSA). The horizontal coordinate is the difference multiplier (log‐transformed with a base of 2) for the fold change (FC), and the vertical coordinate is the *P* value for the difference (log‐transformed with a base of 10). Green dots represent proteins with FC values ≤ screening conditions; red dots, FC values ≥ screening conditions; and black dots, no significant change. (b) Heat map of differential protein expression. Each row in the map represents a protein, each column is a sample/replicate, and different colors indicate different expression levels; red indicates high expression and green indicates low expression. We used log10 values as quantitative values and median correction was applied. This *P* value is not a false discovery rate. (c) Metascape enrichment analyses of the pathways associated with downregulated differentially expressed proteins. The horizontal coordinate Log10 (*p*) is the value of *p* in log base 10; the vertical coordinate is the enriched pathway.

One of the most popular methods for detecting proteins in plasma is LC‐MS/MS. A total of 682 proteins were identified in the current investigation using LC‐MS/MS analysis, and 57 of those proteins were DEPs (Tables S1 and S2). Between patients with RSA and healthy pregnant controls, there were substantial variations in the amounts of these 57 proteins: 21 proteins were considerably elevated, and 36 proteins were significantly downregulated (Figure 1, Table 3). The protein expression patterns of patients with RSA were different from those of healthy pregnant controls, according to a cluster analysis of these DEPs. Among them, we found that the concentration of IGFBP2 was largely decreased in the plasma of RSA patients.

**TABLE 3:** Changes in plasma protein expression for patients with recurrent spontaneous abortion (RSA) compared with healthy pregnant controls.

Change	Protein accession number	Protein symbol	Protein description	Fold change
Increased	Q9NZT1	CALML5	Calmodulin‐like protein 5	49.31793
Increased	P20930	FLG	Filaggrin	27.97923
Increased	P12277	CKB	Creatine kinase	27.06071
Increased	H7BY64	ZNF511‐PRAP1	ZNF511‐PRAP1 readthrough (Fragment)	14.07348
Increased	Q9P232	CNTN3	Contactin‐3	11.83651
Increased	P08670	VIM	Vimentin	10.47412
Increased	Q13885	TUBB2A	Tubulin beta‐2A chain	9.372709
Increased	P02741	CRP	C‐reactive protein	8.527245
Increased	A0A7I2V599	HSPD1	60 kDa heat shock protein, mitochondrial	6.465936
Increased	G3V2Y4	SHMT2	Glycine hydroxymethyltransferase	6.406078
Increased	P54886	ALDH18A1	Delta‐1‐pyrroline‐5‐carboxylate synthase	5.820043
Increased	P04818	TYMS	Thymidylate synthase	5.018111
Increased	P07900	HSP90AA1	Heat shock protein HSP 90‐alpha	3.942921
Increased	Q9UII2	ATP5IF1	ATPase inhibitor, mitochondrial	3.910618
increased	Q9NY15	STAB1	Stabilin‐1	3.760155
Increased	P36980	CFHR2	Complement factor H‐related protein 2	3.257481
Increased	P00338	LDHA	L‐lactate dehydrogenase A chain	2.5679
Increased	A0A590UJ50	CUL1	Cullin‐1	1.93269
Increased	P08709	F7	Coagulation factor VII	1.612481
Increased	Q2KHR2	RFX7	DNA‐binding protein RFX7	1.610155
Increased	A0A096LPE2	SAA2‐SAA4	SAA2‐SAA4 readthrough	1.585009
Decreased	C9IZG4	CUTA	Protein CutA	0.66372
Decreased	Q16853	AOC3	Membrane primary amine oxidase	0.662319
Decreased	Q5TBF5	OGN	Mimecan (Fragment)	0.607444
Decreased	O60216	RAD21	Double‐strand‐break repair protein rad21 homolog	0.600058
Decreased	A2NJV5	IGKV2‐29	Immunoglobulin kappa variable 2–29	0.597405
Decreased	Q99784	OLFM1	Noelin	0.573918
Decreased	H0YCV9	CD44	CD44 antigen (Fragment)	0.553145
Decreased	Q12860	CNTN1	Contactin‐1	0.545997
Decreased	P01602	IGKV1‐5	Immunoglobulin kappa variable 1–5	0.529698
Decreased	Q6YHK3	CD109	CD109 antigen	0.525126
Decreased	Q6UXB8	PI16	Peptidase inhibitor 16	0.470647
Decreased	Q16777	H2AC20	Histone H2A type 2‐C	0.45164
Decreased	P01871	IGHM	Immunoglobulin heavy constant mu	0.395486
Decreased	Q9UFW8	CGGBP1	CGG triplet repeat‐binding protein 1	0.38565
Decreased	P18065	IGFBP2	Insulin‐like growth factor‐binding protein 2	0.274695
Decreased	H9KV31	NCAM2	Neural cell adhesion molecule 2	0.233204
Decreased	P35754	GLRX	Glutaredoxin‐1	0.233043
Decreased	Q8WWA0	ITLN1	Intelectin‐1	0.228822
Decreased	J3KR24	IARS1	Isoleucine–tRNA ligase	0.17517
Decreased	P48426	PIP4K2A	Phosphatidylinositol 5‐phosphate 4‐kinase type‐2 alpha	0.174058
Decreased	P31948	STIP1	Stress‐induced‐phosphoprotein 1	0.159402
Decreased	O00566	MPHOSPH10	U3 small nucleolar ribonucleoprotein protein MPP10	0.153884
Decreased	A0A7P0Z4D3	TNPO1	Transportin‐1	0.131655
Decreased	Q9Y6X0	SETBP1	SET‐binding protein	0.12996
Decreased	E7ETR0	RUVBL1	RuvB‐like helicase	0.128957
Decreased	H7C463	IMMT	MICOS complex subunit MIC60	0.1191
Decreased	H0YN14	IPO4	Importin‐4	0.118492
Decreased	P00441	SOD1	Superoxide dismutase [Cu‐Zn]	0.11709
Decreased	P01718	IGLV3‐27	Immunoglobulin lambda variable 3–27	0.098263
Decreased	Q9Y2W1	THRAP3	Thyroid hormone receptor‐associated protein 3	0.09706
Decreased	Q9HBR0	SLC38A10	Putative sodium‐coupled neutral amino acid transporter 10	0.096692
Decreased	A0A0C4DH38	IGHV5‐51	Immunoglobulin heavy variable 5–51	0.083607
Decreased	A0A0B4J1U7	IGHV6‐1	Immunoglobulin heavy variable 6–1	0.083014
Decreased	Q13449	LSAMP	Limbic system‐associated membrane protein	0.077074
Decreased	Q05639	EEF1A2	Elongation factor 1‐alpha 2	0.030081
Decreased	P62841	RPS15	40S ribosomal protein S15	0.007854

*Note*: Fold change is the ratio of the protein concentrations between the samples collected from healthy pregnant controls and from patients with RSA.

### 3.3 GO analysis

Through GO functional annotation analysis of the obtained experimental data, we found that for the GO category of biological processes, “cellular process” was linked to the highest percentage of DEPs (*n* = 43 proteins; Figure S3). The three most upregulated proteins in this cluster were CALML5, FLG, and CKB, while the three most downregulated proteins were AOC3, RAD21, and OLFM1. For the GO category of cellular component, the highest percentage of DEPs was associated with the term “cellular anatomical entity” (*n* = 50). The top three upregulated proteins in this cluster were VIM, HSP90AA1, and ATP5IF1, while the top three proteins that were downregulated were AOC3, RAD21, and IGKV2. For the GO category of molecular function, the highest percentage of DEPs was associated with the term “binding” (*n* = 49). The top three elevated proteins in this cluster were CALML5, FLG, and CKB, while the top three downregulated proteins were AOC3, OGN, and RAD21.

According to our GO analysis, the enriched upregulated DEPs were primarily connected to the functional word “binding,” and the functional words “nuclear protoplasm,” “nucleus,” and “nucleic acid binding” were mostly linked to the downregulated DEPs (Figure S4). In our GO enrichment analysis, we found four proteins among the downregulated proteins related to proliferation: IGFBP2, SOD1, OLFM1, and PI16. Numerous research have demonstrated that IGFBP2 is crucial for controlling cell proliferation. As a result, the current research concentrated on conducting additional tests to evaluate the mechanisms underlying IGFBP2's actions.

### 3.4 Metascape enrichment analysis

Metascape is for annotating and analyzing gene lists. It combines functional enrichment, interaction group analysis, and gene annotation (Zhou et al., 2019). The results of our Metascape analysis of the downregulated proteins indicated an enrichment of the following pathways: negative regulation of catalytic activity, regulation of developmental growth, cellular responses to stress, ribonucleoprotein complex biogenesis, and positive regulation of organelle organization (Figure 1). We also found pathways that regulate cell proliferation.

### 3.5 Expression change of IGFBP2 in trophoblasts of decidual tissues from RSA patient

**FIGURE 2:**
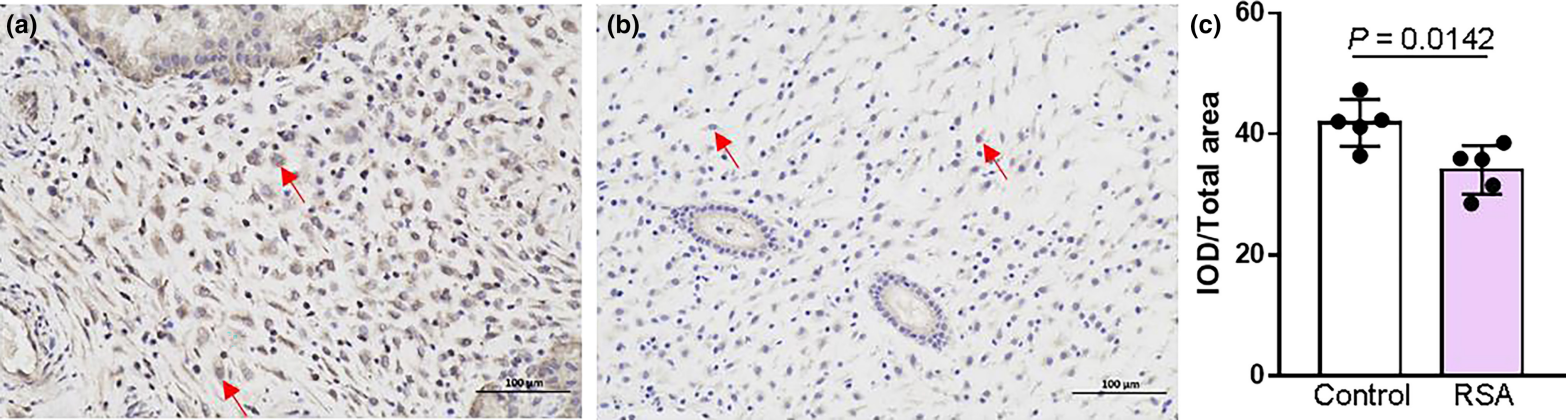
Trophoblast IGFBP2 expression in decidual tissues from healthy women with normal pregnancies and recurrent spontaneous abortion (RSA) patient. (a–c) Representative images (a and b) and summary data (c) showing the expression of IGFBP2 in the trophoblasts indicated by arrows of decidual tissues from healthy women with normal pregnancies and RSA patient. Data showing the ratio of integrated optical density (IOD) divided by total area. The scale bar in (a and b) is 200 μm. Data are shown as the mean ± SD; *n* = 5.

There are many trophoblasts in the decidual tissues. To investigate IGFBP2 protein expression change in the trophoblast of the decidual tissues from RSA patient, we collected clinical decidual tissues from healthy women with normal pregnancies and RSA patients. Our immunohistochemical data showed that the expression level of IGFBP2 protein was significantly decreased in the trophoblasts of the decidual tissues from RSA patients compared to that from healthy women with normal pregnancies (Figure 2). This finding is consistent with the result of plasma IGFBP2 concentration change examined by LC‐MS/MS experiments.

### 3.6 Effect of IGFBP2 on proliferation

**FIGURE 3:**
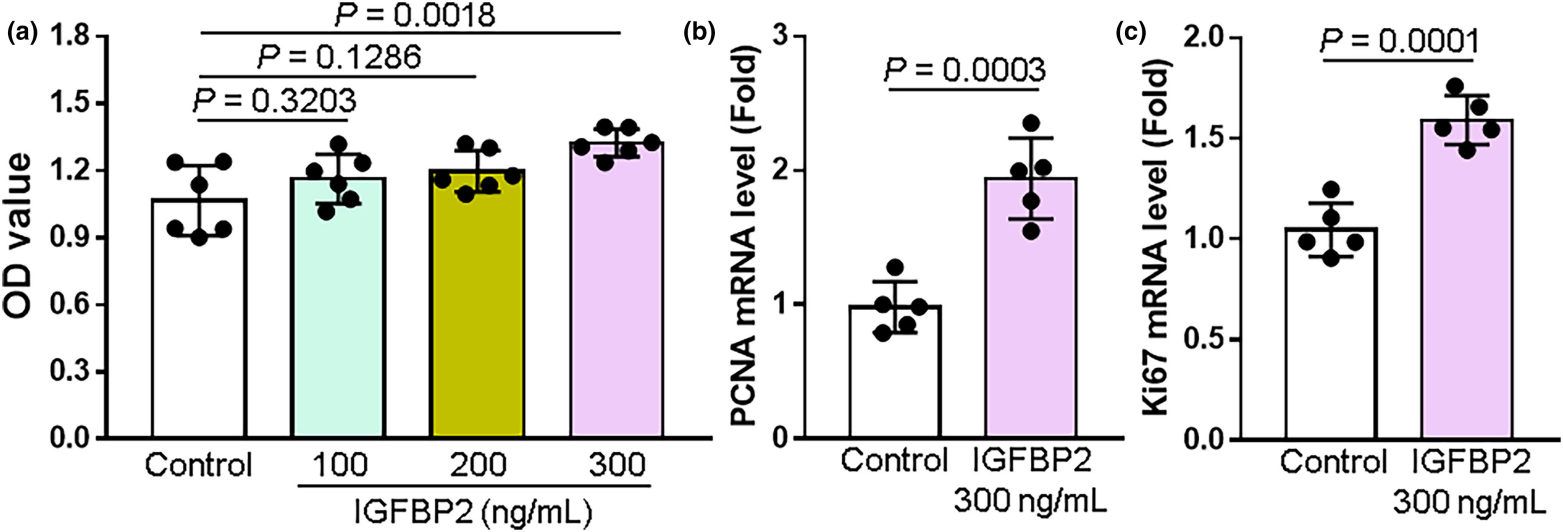
Application of IGFBP2 increases HTR‐8/SVneo cell proliferation. (a) A CCK8 assay was used to assess the viability of cells cultured with or without increasing concentrations (100, 200, 300 ng/mL) of IGFBP2 for 48 h (*n* = 5). OD represents relative optical density. (b–c) Summary data showing relative mRNA expression levels of proliferating cell nuclear antigen gene (*PCNA*) and *Ki67* of HTR‐8/SVneo cells treated with or without IGFBP2 (300 ng/mL, *n* = 5). The mRNA levels were normalized to 18S rRNA. Data represent the mean ± SD.

After treating HTR‐8/SVneo cells with different concentrations (100, 200, and 300 ng/mL) of IGFBP2 for 48 h, we measured cell proliferation using CCK8 assay kits. High doses of IGFBP2 increased the proliferation of HTR‐8/SVneo cells compared to the control group (Figure 3a). In addition, IGFBP2 (300 ng/mL) treatment for 24 h significantly increased HTR‐8/SVneo cell mRNA expressions of proliferating cell nuclear antigen (PCNA) and Ki67, both which are biomarkers for cell proliferation (Figure 3b,c).

### 3.7 Effect of IGFBP2 on transcript expression

**FIGURE 4:**
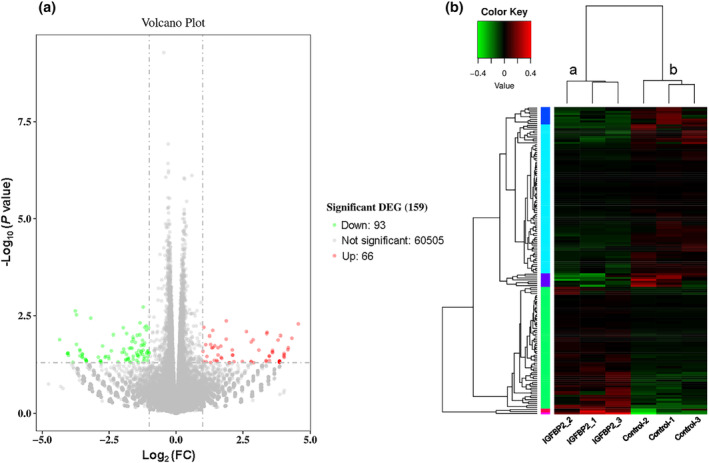
Volcano plot and heat map of genes differentially expressed in trophoblast following treatment with IGFBP2. (a) Volcano plot of the number of differentially expressed genes (DEGs). FC represents fold change. Red dots represent DEGs that were upregulated; green dots, downregulated DEGs. (b) Cluster map of DEGs. The log10 (FPKM+1) values were used for clustering. Color ranges from red, indicating genes that were highly expressed, to green, indicating genes with low expression. Control group: Control‐1‐3; IGFBP2‐treated group: IGFBP2‐1‐3 (300 ng/mL of IGFBP2 for 48 h).

A cluster analysis showed that mRNAs were differentially expressed in trophoblast cells treated with IGFBP2 compared with untreated cells. 66 genes were upregulated and 93 genes were downregulated out of 159 genes with substantially altered expression (Table S3). The differentially expressed transcripts are illustrated in a volcano plot and heat map (Figure 4). In IGFBP2‐treated trophoblast cells, the top 10 differentially expressed genes (DEGs) with upregulated expression were RPL5P18, TMEM45B, LUM, KRTAP4‐11, ELANE, RNU6‐623P, KRTAP5‐7, RNU6‐925P, LINC02167, and CASC11; and the top 10 downregulated DEGs were TRIM60P17, RNF138P1, MIR5188, TNFRSF10C, KLHL32, CABP4, MUC5B, RARRES2P2, DSP, and GATA6‐AS1.

The top 30 terms resulting from a GO enrichment analysis reflecting the distribution of the DEGs among three GO terms (biological process, cellular component, and molecular function) are shown in Figure S5. The highest percentage of DEGs in the classification of biological process was associated with the term “visual perception” (*n* = 4 genes). The two upregulated genes in this cluster were RPGRIP1 and LUM, while the two downregulated genes were OPN1SW and CABP4. In the classification of cellular component, the highest percentage of DEGs was associated with the term “integral component of the presynaptic membrane” (*n* = 2). No genes were upregulated, and the two downregulated genes were GRIK5 and ADAM23. For the classification of molecular function, the highest percentage of DEGs was associated with the term “calcium channel regulator activity” (*n* = 2). No genes were upregulated, and the two downregulated genes were REM2 and CABP4.

**FIGURE 5:**
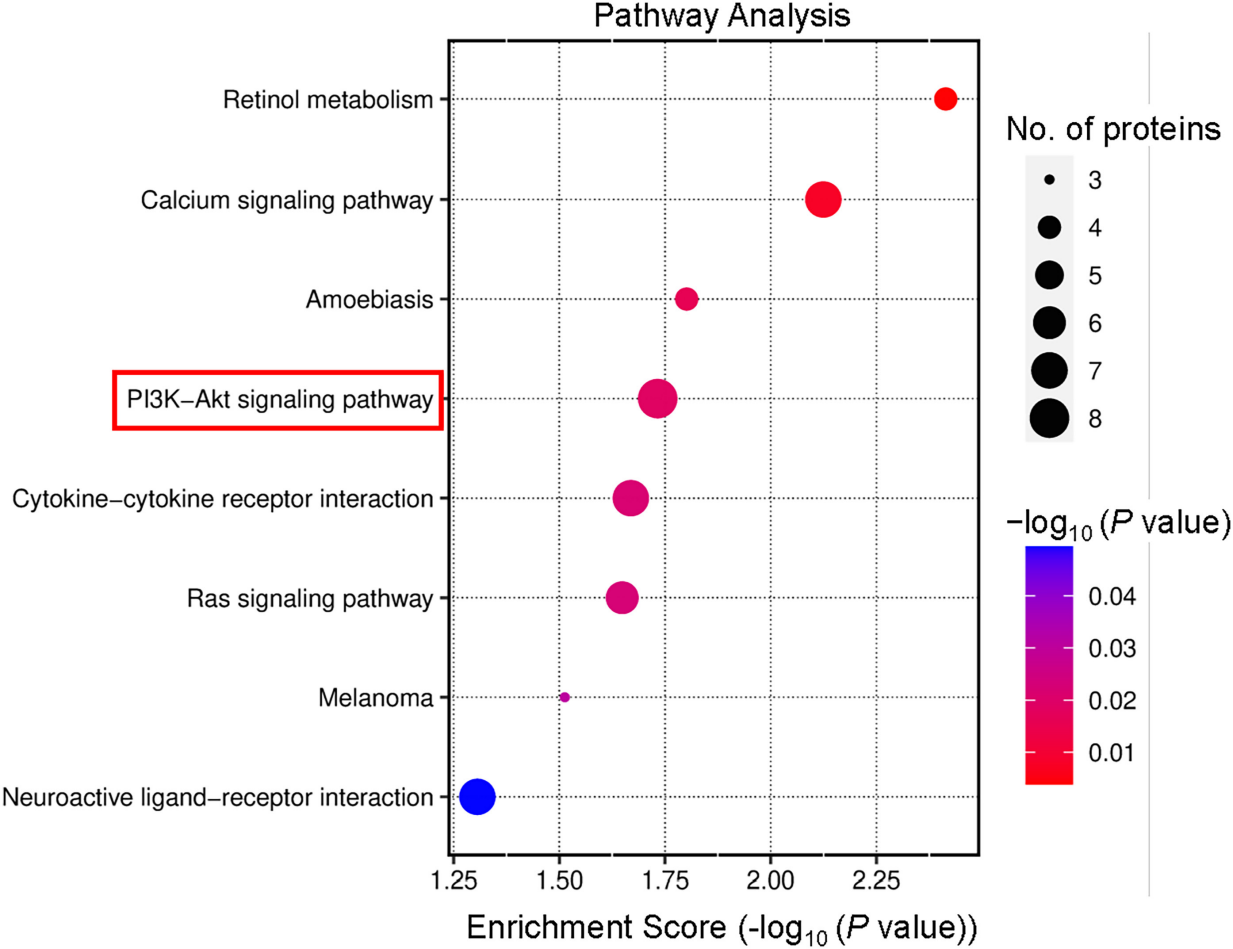
Pathway enrichment for genes with variable expression in Kyoto Encyclopedia of Genes and Genomes (KEGG) database. The ratio of the proportion of genes found in the classification times the total proportion of differentially expressed genes in the KEGG signaling pathway represents the enrichment factor, which is indicated by the horizontal coordinate. KEGG word definitions are listed in the vertical coordinates. *p* value is the *p* value of the enrichment obtained using Fisher's exact test; −log10 (*p* value) is the log‐transformation of the Fisher's exact test *p* value. Bubble size reflects the number of differentially expressed proteins in the KEGG pathway. *p* Value represents the unadjusted false discovery rate.

A KEGG analysis was used for the upregulated genes. The upregulated annotated KEGG pathways were retinol metabolism, calcium signaling pathway, amebiasis, PI3K‐Akt signaling pathway, Ras signaling pathway, cytokine‐cytokine receptor interaction, melanoma, and neuroactive ligand‐receptor interaction (Figure S6). An enrichment analysis of the KEGG pathways showed that the main pathways associated with IGFBP2 were the PI3K‐Akt signaling pathway, cytokine‐cytokine receptor interaction, neuroactive ligand‐receptor interaction, and the calcium signaling pathway (Figure 5). The DEGs associated with the PI3K‐Akt signaling pathway were HGF, LPAR4, AREG, CSF3R, IGF2, FGF23, FGF18, GNG8. The DEGs associated with cytokine‐cytokine receptor interaction were CCR1, MSTN, CSF3R, IL17C, EDA, CCL5, and IL37. The DEGs associated with neuroactive ligand‐receptor interactions were F2RL2, LPAR4, GRIK4, TAC3, NMBR, GNRHR, and NTS. The DEGs associated with the calcium signaling pathway were HGF, GNA14, SLC25A31, TRDN, FGF23, CD38, and FGF18.

**FIGURE 6:**
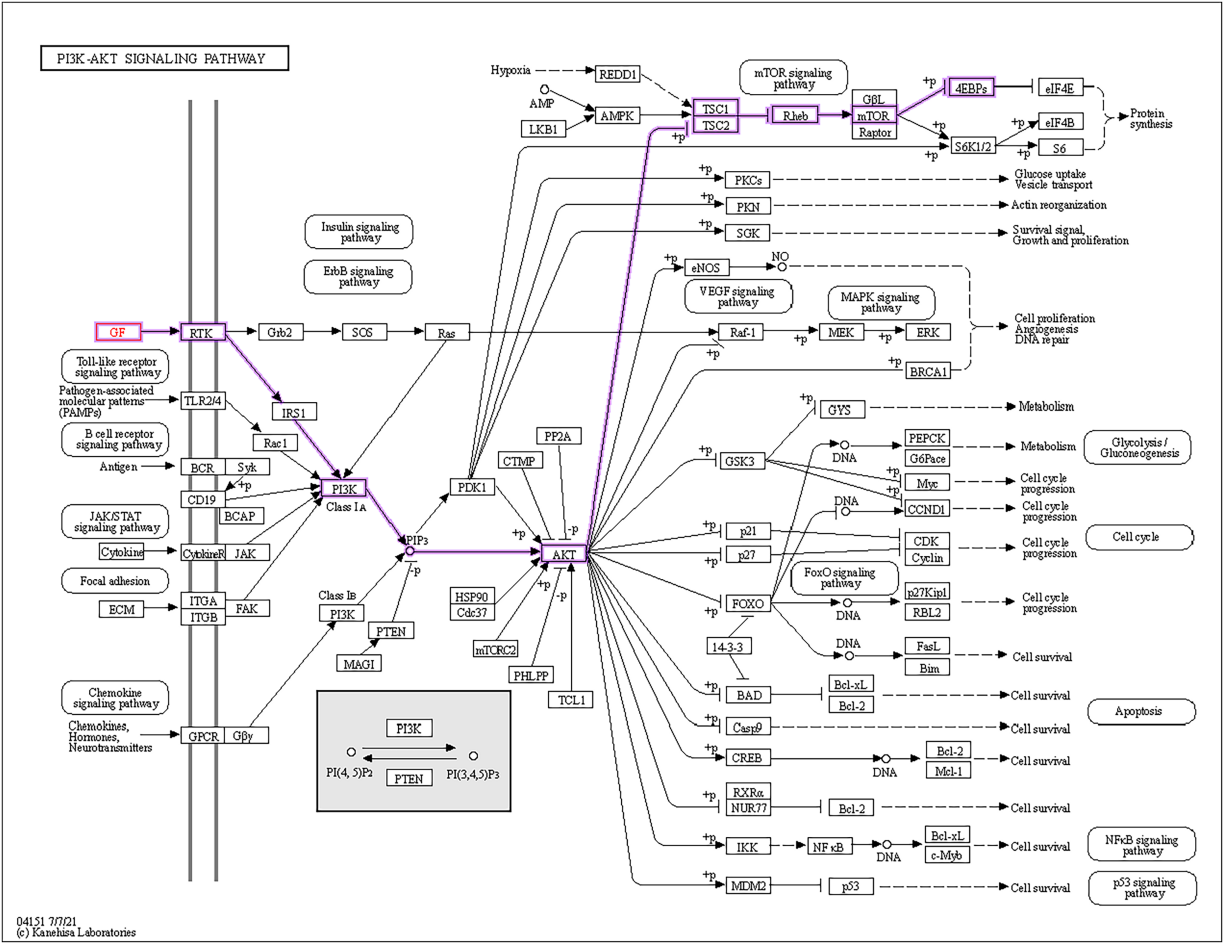
IGF–IGF‐1R–PI3K signaling pathway.

Among these DEGs, IGF2 is closely related to IGFBP2. IGFBP2 is one of six IGF‐binding proteins in the human body (IGFBP1–6), which have high affinity for binding IGF1 and IGF2 (Yu & Rohan, 2000). Therefore, we speculate that IGFBP2 regulates trophoblast proliferation through the PI3K‐Akt signaling pathway. The interaction of IGF and its receptor with the PI3K‐Akt signaling pathway is mapped in Figure 6. The KEGG map was permitted to publish by Kanehisa Laboratories.

### 3.8 IGF1R‐PI3K‐Akt signaling pathway has a role in IGFBP2‐induced proliferation

**FIGURE 7:**
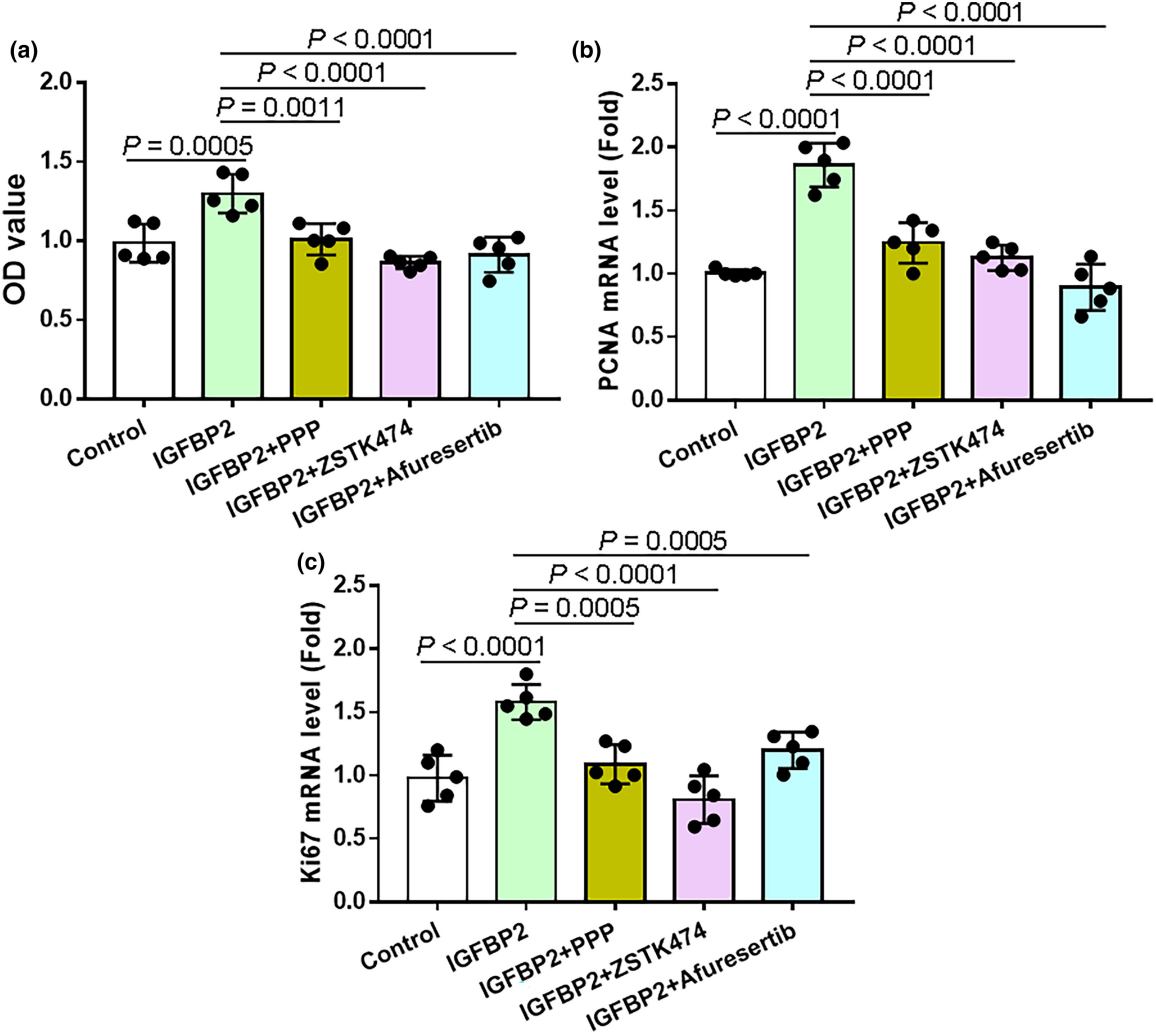
Effect of IGF1R‐PI3K‐Akt signaling pathway inhibitors on IGFBP2‐induced proliferation of HTR‐8/SVneo cells. Summary data showing cell viability (a, CCK8 assay) and relative mRNA expression levels (b–c) of proliferating cell nuclear antigen gene (*PCNA*) and *Ki67* of HTR‐8/SVneo cells treated with solvent control (Control), 300 ng/mL IGFBP2 or IGFBP2 + picropodophyllin (PPP, 0.2 μM), IGFBP2 + ZSTK474 (0.1 μM, PI3K inhibitor), IGFBP2 + afuresertib (0.2 μM, Akt inhibitor) for 48 h (*n* = 5). OD represents relative optical density. The mRNA levels were normalized to 18S rRNA. Data represent the mean ± SD.

It is known that IGFBP2 mainly acts on IGF‐1 receptor (IGF1R) to mediate signal transduction (Mazerbourg et al., 2003). Therefore, we used IGF1R and PI3K‐Akt signaling pathway inhibitors to identify the role of IGF1R‐PI3K‐Akt signaling pathway in IGFBP2‐induced proliferation of trophoblast cells. Our data indicated that picropodophyllin (PPP, 0.2 μM) as an inhibitor of IGF1R significantly abolished IGFBP2‐induced proliferation of HTR‐8/SVneo cells (Figure 7a). PI3K inhibitor ZSTK474 (0.1 μM) and Akt inhibitor afuresertib (0.2 μM) also significantly inhibited IGFBP2‐induced proliferation of HTR‐8/SVneo cells (Figure 7a). In addition, PPP, ZSTK474, and afuresertib also significantly inhibited IGFBP2‐increased mRNA expressions of PCNA and Ki67 of HTR‐8/SVneo cells (Figures 7b,c).

## 4 DISCUSSION

**FIGURE 8:**
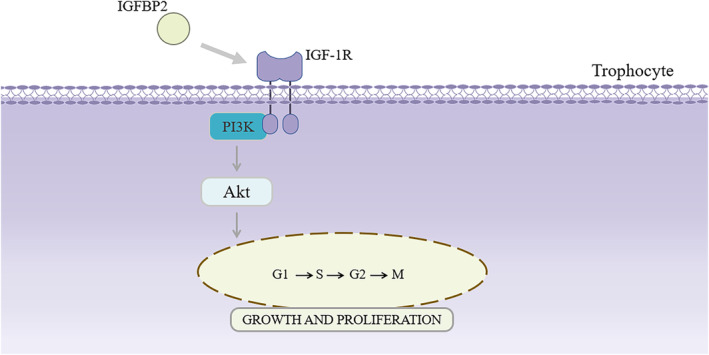
Schematic diagram of the mechanism through which IGFBP2 may regulate cell proliferation via the PI3K‐Akt signaling pathway. IGFBP2 activates IGF‐1 receptor (IGF‐1R) on the trophoblast plasma membrane, further activating actions downstream of the PI3K‐Akt signaling pathway to regulate cell growth and proliferation.

Around 1% of pregnant women are affected by RSA, which has a detrimental effect on patients' physical and emotional health. RSA may be caused by congenital malformations, blood clotting disorder, metabolic disturbances, anatomical variables, and immune reactions (Horne & Alexander, 2005). Yet, the primary molecules involved in these intricate disease processes remain unclear. Among the numerous molecules possibly involved, inflammation conditions related to an impaired expression of alpha1‐antitrypsin might affect the embryo formation, thus increasing the possibilities of adverse pregnancy outcome (Rotondo et al., 2020). In this study, using label‐free and LC‐MS/MS analyses, we identified plasma DEPs between healthy pregnant women and patients with RSA. Of 682 proteins identified, 57 were DEPs: a substantial downregulation of 36 and an upregulation of 21. In GO enrichment analyses of the DEPs, we identified four that were related to cell proliferation: IGFBP2, SOD1, OLFM1, and PI16. To confirm these findings from the bioinformatics analysis using proteomics, we performed cell‐based experiments. The results of trophoblast cell proliferation experiments and transcriptome sequencing analyses indicated that, via the PI3K‐Akt signaling pathway, IGFBP2 may contribute to the proliferation of trophoblast cells (Figure 8).

IGFBP2, a crucial member of the IGFBP family and a 36‐kDa protein that is highly produced throughout fetal development, has been recognized as a significant oncogene (Shimasaki & Ling, 1991; Wei et al., 2021). IGFBP2 and the functionality of cancer cells are intimately connected, according to numerous studies (Prayudi et al., 2020; Sun et al., 2021; Tan et al., 2023; Tang et al., 2018; Zheng et al., 2020; Zhu et al., 2019). The heparin‐binding domain 1 of matured IGFBP2 is required for IGFBP2‐mediated cell proliferation, migration, and invasion (Arai et al., 1996; Li et al., 2020; Russo et al., 1997, 1999, 2005). In addition, IGFBP2 is an IGF system regulator that controls the distribution, function, and activity of IGFs in the pericellular space (Firth & Baxter, 2002; Pereira et al., 2004; Russo et al., 1999; Schutt et al., 2004).

The trophoblast is a key component of placental and fetal development during early conception (Zong et al., 2016). Successful trophoblastic migration and penetration of maternal tissue are necessary for a healthy placenta and pregnancy maintenance (Miao et al., 2020). Previous studies have shown that severe depletion of the trophoblast stem cell pool caused by apoptosis or premature differentiation will affect placental morphogenesis and may lead to early abortion and pregnancy‐related complications (Watson & Cross, 2005). According to these previous findings from other groups, trophoblast proliferation is crucial to the occurrence and progression of pregnancy. In our study, application of IGFBP2 enhanced trophoblast cell proliferation, suggesting that IGFBP2 is a crucial regulatory factor in regulating trophoblast cell growth. Transcriptome sequencing suggested that the PI3K‐Akt signaling pathway in trophoblast cells treated with IGFBP2 was significantly elevated. In addition, several studies have also found that the PI3K‐Akt signaling pathway is important in cell proliferation and is linked to IGFBP2 (Mehrian‐Shai et al., 2007; Mireuta et al., 2010; Wilhelm et al., 2015; Yan et al., 2021). Therefore, we examined the role of PI3K‐Akt signaling pathway in IGFBP2‐induced trophoblast cell proliferation next. Our results showed that inhibitors of IGF1R‐PI3K‐Akt signaling pathway also significantly abolished IGFBP2‐induced trophoblast cell proliferation. Thus, IGFBP2 may act on IFG1R to stimulate the PI3K‐Akt pathway to regulate trophoblast proliferation. In the present study, the number of clinical samples is limited. Therefore, in order to validate the study findings, the plasma levels of IGFBP2 should be evaluated in a larger cohort of RSA females compared to healthy females in the future.

Reactive oxygen species (ROS) are closely associated with a number of physiological and pathological functions and may influence the function of trophoblast and outcome of pregnancy (Dos Anjos Cordeiro et al., 2023; Ye et al., 2023). More recently, Ray et al. reported that the single nucleotide polymorphisms and expression level of SOD1 and SOD2 are associated with C. trachomatis‐infected recurrent spontaneous abortion RSA (Ray et al., 2022, 2023). These studies indicate that SOD1 may be an important potential factor related to RSA. In our data from label‐free and LC‐MS/MS analyses show that SOD1 concentration in the plasma of RSA patients is significantly lower than healthy control. Therefore, our study also provides the potential role of SOD1 in the occurrence and development of RSA. However, the detailed molecular mechanism for SOD1 in the function of trophoblast and outcome of pregnancy may be investigated in future.

Even we provide some valuable information for the possible mechanism of RSA through LC‐MS/MS analyses and bioinformatics, there are some limitations in the present study. Because the clinical samples are very hard to be obtained, our sample sizes are very small. In addition, some results found from proteomics analysis should be confirmed by cell‐based and/or animal experiments. The future further study is useful to confirm our findings.

## 5 CONCLUSION

This study leveraged proteomics techniques to identify and quantify the evolution of important plasma proteins and possible contributions to the pathophysiology of RSA. One of our key findings was that in RSA patients, IGFBP2 expression was downregulated. The results of trophoblast cell proliferation experiments and transcription sequencing experiments suggested that IGFBP2 has the potential to influence pregnancy outcomes by regulating trophoblast proliferation via the PI3K‐Akt signaling pathway. Thus, IGFBP2 may be a potential biomarker and therapeutic target for RSA. This study points the way forward for the diagnosis and treatment of RSA.

## FUNDING INFORMATION

This research was funded by a National Natural Science Foundation of China (grant No. 82060882), a National Natural Science Foundation of China Regional Innovation and Development Joint Fund (grant No. U22A20272) and a FDCT grant from the Macao Science and Technology Development Fund (grant code: 002/2023/ALC).

## CONFLICT OF INTEREST STATEMENT

The authors declare that the research was conducted in the absence of any commercial or financial relationships that could be construed as a potential conflict of interest.

### ETHICS APPROVAL STATEMENT

The present study was approved by Lu’an Traditional Chinese Hospital Medical Ethics Committee (2021‐KY‐LL‐001) in accordance with the Declaration of Helsinki. All participants provided written informed consent.

## Supporting information


Figures S1–S6.

Tables S1–S3.

## Data Availability

The data presented in the study are deposited in the GEO repository, accession number SUB12864236.
