# Supplementary material for: Role of increased IGFBP2 in trophoblast cell proliferation and recurrent spontaneous abortion development: A pilot study
Source: Physiol Rep. 2024 Feb 5;12(3):e15939. doi: 10.14814/phy2.15939 (PMC10843903; doi:10.14814/phy2.15939)
Supplement: Supplementary file 1 — Figures S1–S6. [file PHY2-12-e15939-s002.docx]

**Supplementary Figures**

**
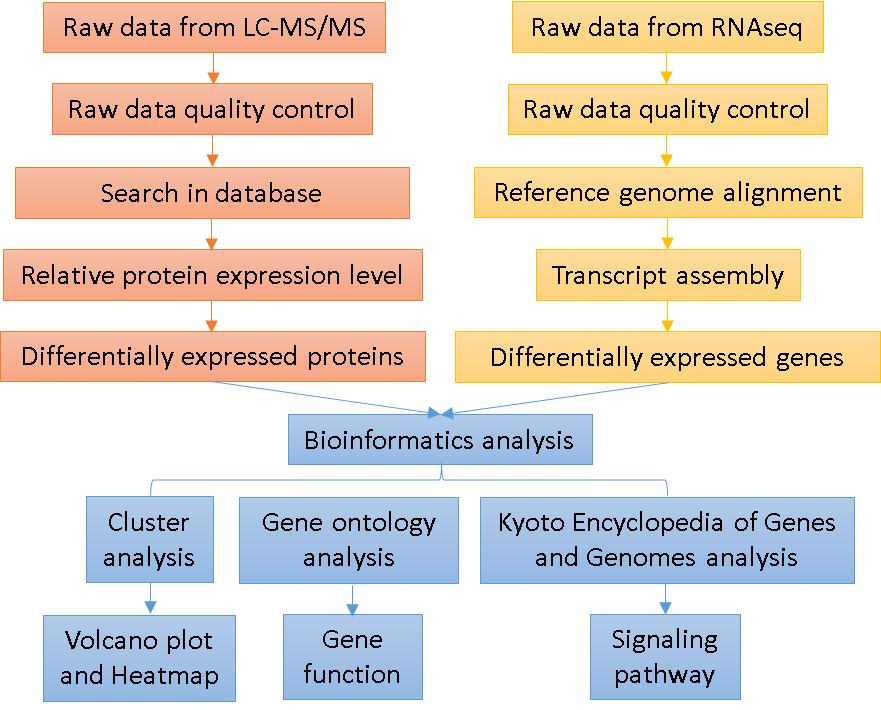
**

**Supplementary Figure 1.** Flowchart of study analyses for LC-MS/MS and RNAseq.


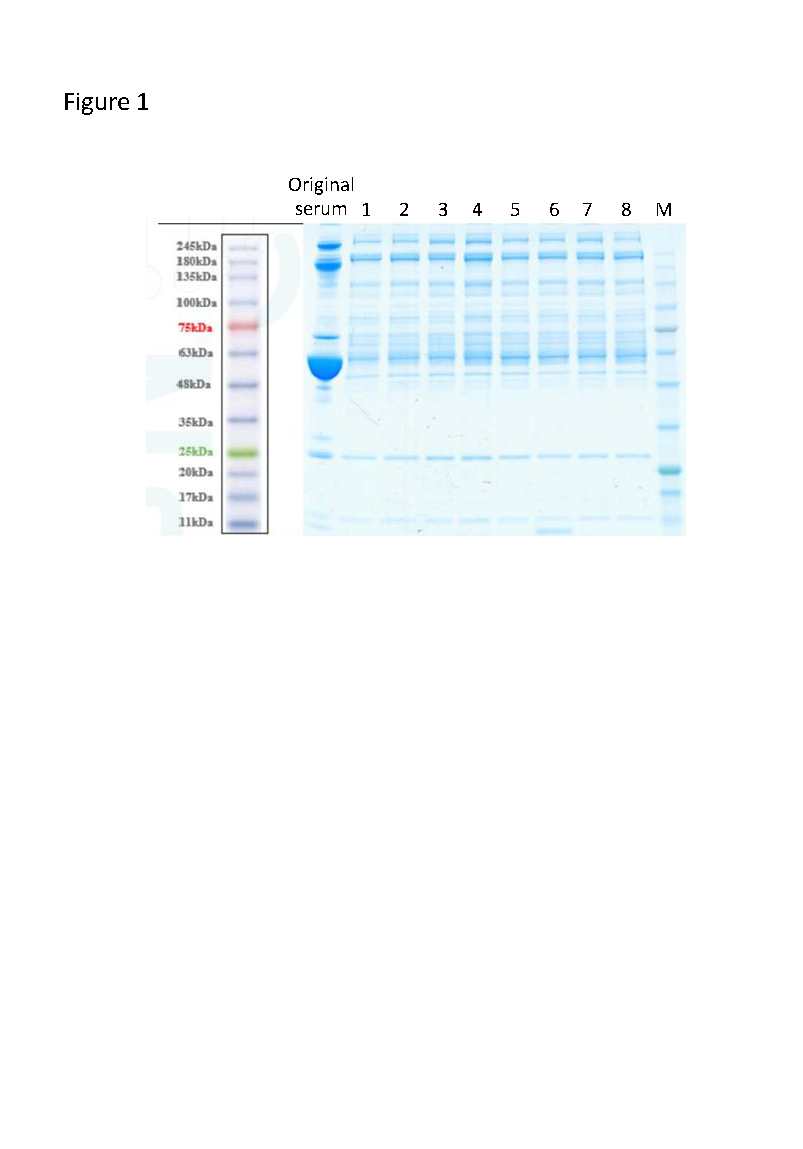


**Supplementary Figure 2. Protein separation and quality control determined by sodium dodecyl sulfate–polyacrylamide gel electrophoresis.** Lane M shows protein markers. The first lane shows the distribution of the proteins in the original plasma sample. Lanes 1–4 are one each of the four protein samples after the highly abundant proteins have been removed from plasma samples of each patient with RSA; lanes 5–8 are one each of the four protein samples after the highly abundant proteins have been removed from plasma samples of four healthy pregnant controls. Compared with the bands in the original plasma, the levels of the high-abundance proteins in lanes 1–8 were significantly decreased, whereas the protein bands in the other molecular weight regions were significantly increased. The amount of total protein met the requirements for further experimental use of the plasma samples.


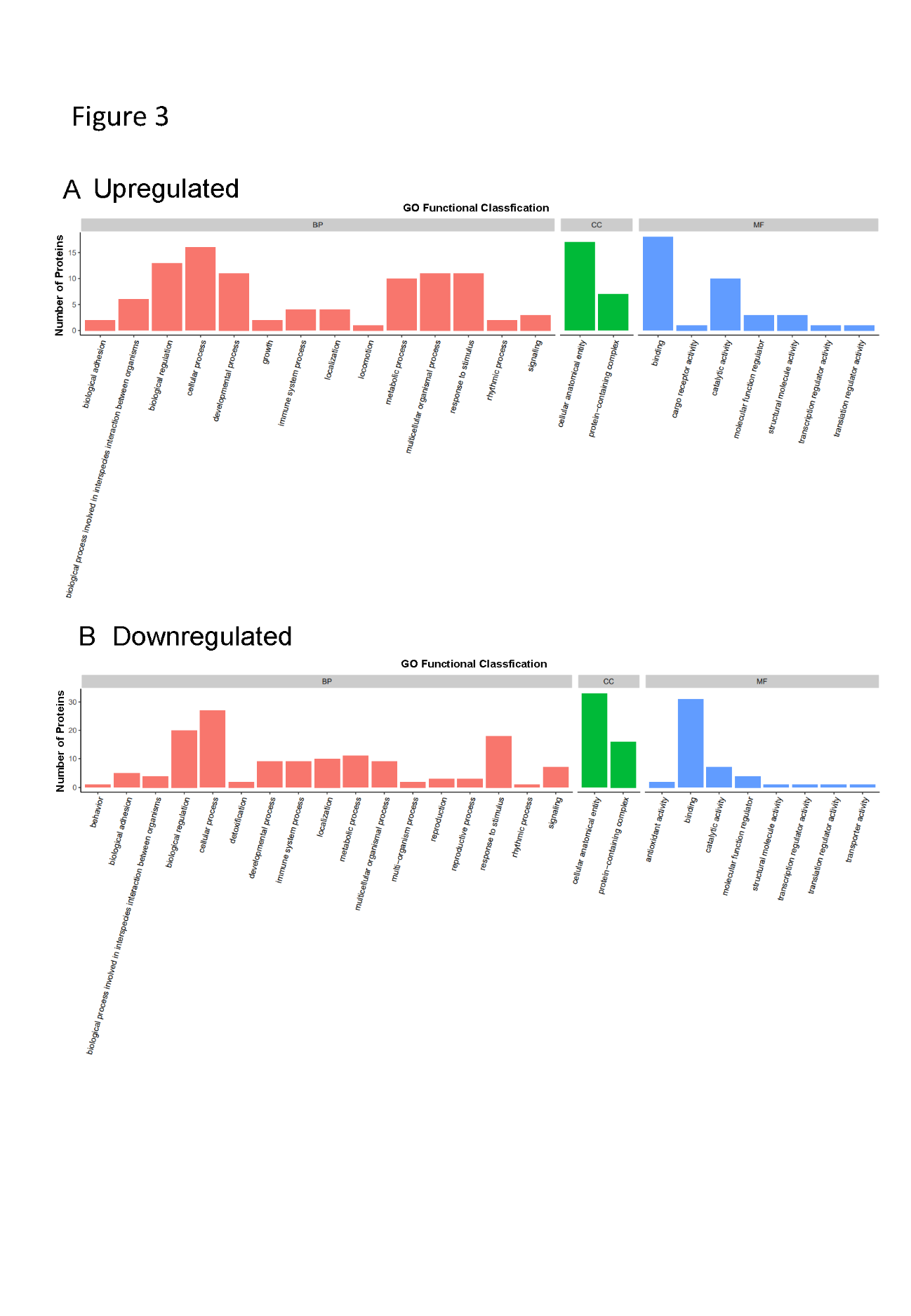


**Supplementary Figure 3. Gene Ontology (GO) annotation for functional categorization.** The horizontal coordinate indicates the term identified for (GO) each GO classification, and the vertical coordinate indicates the number of differentially expressed proteins. BP represents biological process; CC, cellular component; and MF, molecular function.


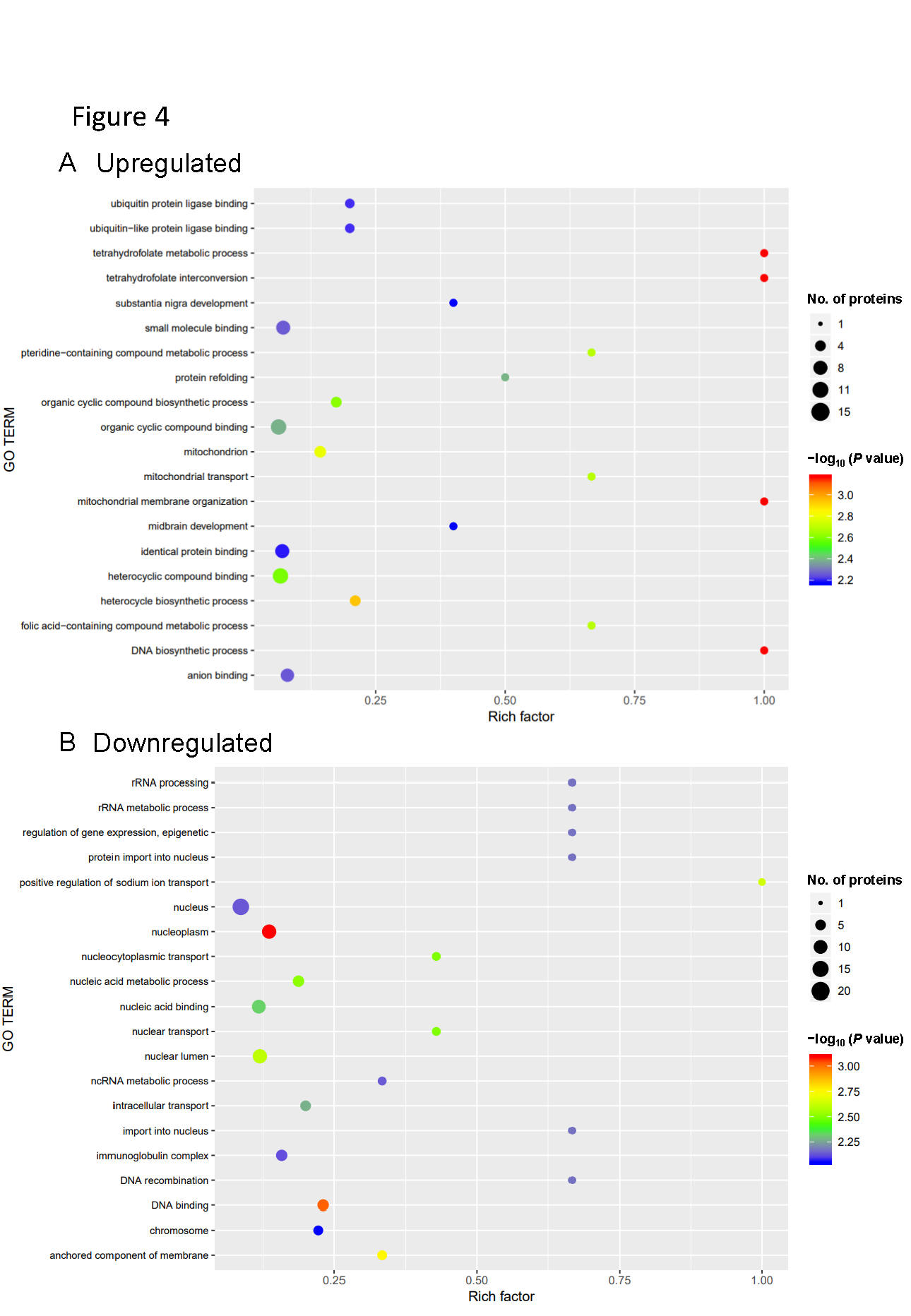


**Supplementary Figure 4. Gene Ontology (GO) enrichment for functional terms.** The horizontal coordinate represents the rich factor: the ratio of the number of differentially expressed proteins in the GO classification relative to the number of identified proteins in the classification. The algorithm is as follows: if Differ = a; Background = b; All Differ = c; All Background = d; then rich factor = a/b. The ordinate is the GO term description (i.e., a detailed description of the GO classification). The size of the bubble indicates the number of differentially expressed proteins in that GO classification. The *P* value is the *P* value of the enrichment obtained using the hypergeometric distribution test; and -log10 (*P* value) is the logarithmic conversion of that *P* value.


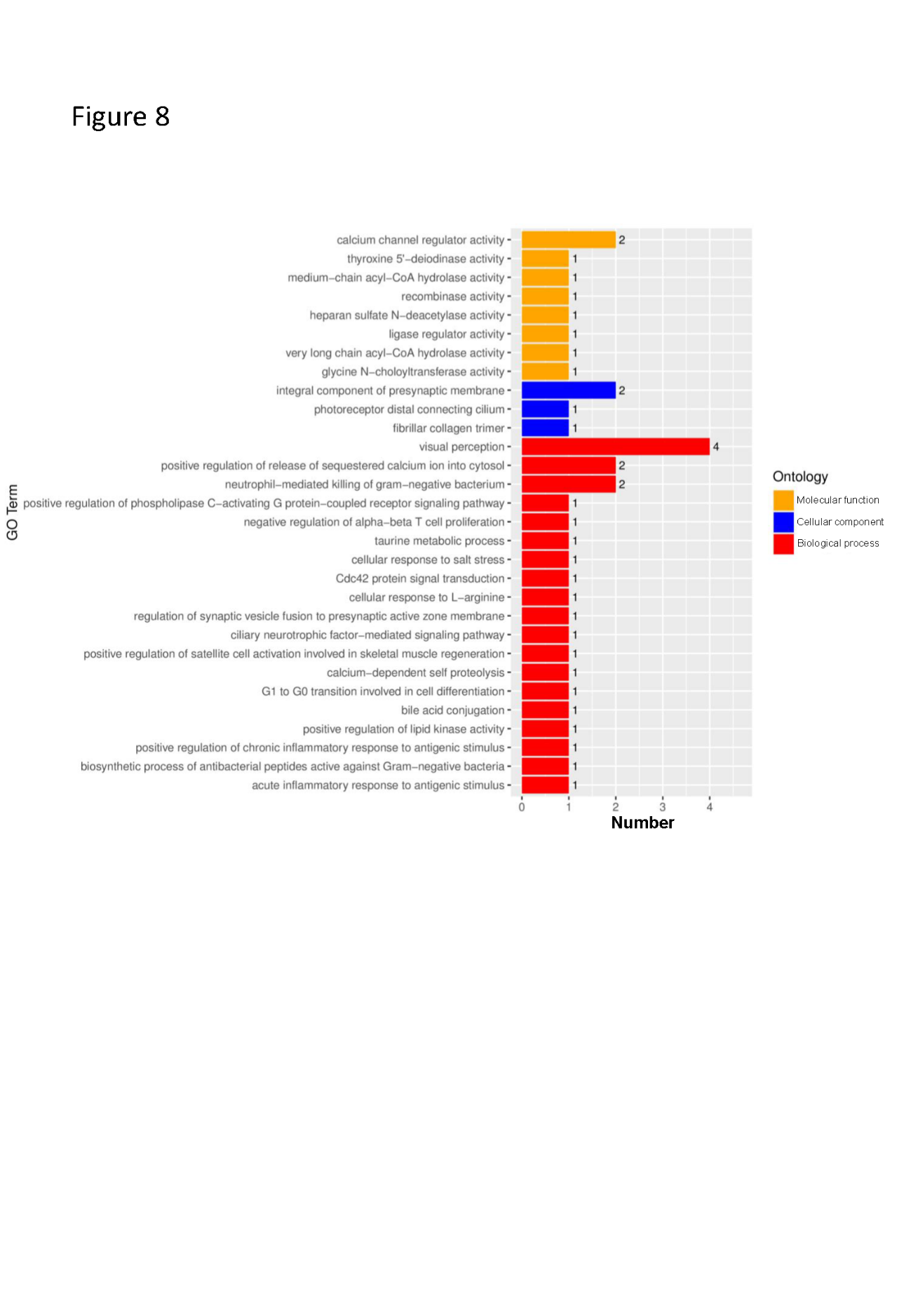


**Supplementary Figure 5. Gene Ontology (GO) enrichment histogram.** The vertical coordinate is the enriched GO term, and the horizontal coordinate is the number of differentially expressed genes in the term. Colors are used to distinguish the classifications of biological process, cellular component, and molecular function.


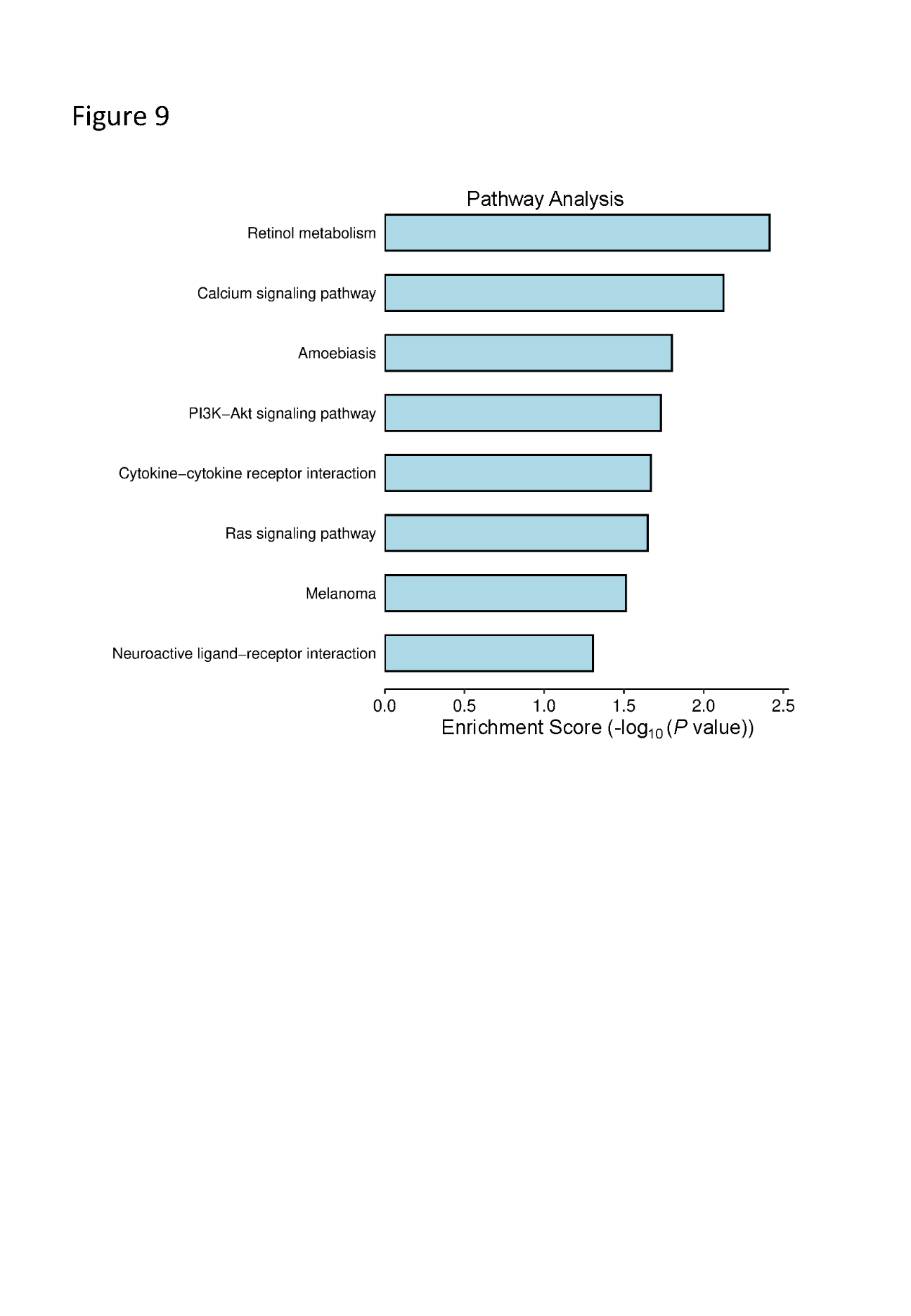


**Supplementary Figure 6. Kyoto Encyclopedia of Genes and Genomes (KEGG) pathway annotations for the differentially expressed genes.** The horizontal coordinate indicates the enrichment factor: the total proportion of differentially expressed genes in the KEGG signaling pathway multiplied by the proportion of genes identified in the classification. The vertical coordinate is the KEGG pathway name.
