# Supplementary material for: Role of increased IGFBP2 in trophoblast cell proliferation and recurrent spontaneous abortion development: A pilot study
Source: Physiol Rep. 2024 Feb 5;12(3):e15939. doi: 10.14814/phy2.15939 (PMC10843903; doi:10.14814/phy2.15939)
Supplement: Supplementary file 2 — Tables S1–S3. [file PHY2-12-e15939-s001.zip › PHYSREP-2023-10-413-s03.pdf]

Accessior	Descripti	Exp. q-va	Protein F	Sum PEP S	Coverage	# Peptide	# PSMs	# Unique
Q9NZT1	Calmoduli	0	High	39.497	53	6	9	6
P20930	Filaggrir	0.003	High	9.895	0	3	4	3
P12277	Creatine	0	High	26.935	13	2	66	2
H7BY64	ZNF511-PF	0.01	High	2.983	3	1	1	1
Q9P232	Contactir	0.011	Medium	2.601	1	1	4	1
P08670	Vimentin	0	High	13.096	9	2	12	1
Q13885	Tubulin b	0	High	39.95	10	4	34	4
P02741	C-reactiv	0	High	20.839	23	6	23	6
A0A7I2V59	60 kDa ch	0	High	14.658	10	2	4	2
G3V2Y4	Glycine h	0	High	10.75	7	1	1	1
P54886	Delta-1-p	0.002	High	5.812	2	1	2	1
P04818	Thymidyla	0.01	High	3.344	7	1	2	1
P07900	Heat shoc	0	High	39.279	10	6	155	6
Q9UII2	ATPase ir	0.004	High	4.253	9	1	6	1
Q9NY15	Stabilin-	0.034	Medium	1.985	0	1	1	1
P36980	Complemer	0	High	27.506	21	5	159	2
P00338	L-lactate	0	High	44.142	26	6	34	5
A0A590UJ5	Cullin-1	0.02	Medium	2.266	4	1	6	1
P08709	Coagulati	0	High	28.038	13	4	25	4
Q2KHR2	DNA-bindi	0.03	Medium	2.005	1	1	2	1
A0A096LPE	SAA2-SAA4	0	High	42.027	28	6	211	5
C9IZG4	Protein C	0.02	Medium	2.445	10	1	4	1
Q16853	Membrane	0	High	17.584	7	4	20	4
Q5TBF5	Mimecan (	0.022	Medium	2.176	3	1	4	1
O60216	Double-st	0.051	Low	1.74	2	1	2	1
A2NJV5	Immunoglo	0.003	High	8.726	20	2	17	1
Q99784	Noelin OS	0	High	22.052	11	4	18	4
H0YCV9	CD44 anti	0	High	20.843	10	3	59	3
Q12860	Contactir	0	High	22.903	8	7	18	7
P01602	Immunoglo	0.004	High	4.246	9	1	8	1
Q6YHK3	CD109 ant	0	High	19.908	5	6	19	6
Q6UXB8	Peptidase	0	High	31.758	11	5	46	5
Q16777	Histone H	0.009	High	3.864	15	1	5	1
P01871	Immunoglo	0	High	118.766	36	15	439	15
Q9UFW8	CGG tripl	0.002	High	5.234	7	1	24	1
P18065	Insulin-l	0	High	36.722	27	7	20	7
H9KV31	Neural ce	0.013	Medium	2.578	2	1	1	1
P35754	Glutaredo	0.01	High	3.102	10	1	1	1
Q8WWA0	Intelecti	0.009	High	2.778	3	1	1	1
J3KR24	Isoleucyl	0.056	Low	1.652	3	1	2	1
P48426	Phosphati	0.002	High	5.786	3	1	1	1
P31948	Stress-ir	0.025	Medium	2.088	1	1	2	1
O00566	U3 small	0.009	High	3.653	3	1	1	1
A0A7P0Z4I	Transport	0.009	High	3.787	2	1	3	1
Q9Y6X0	SET-bindi	0.05	Low	1.843	2	1	1	1
E7ETR0	RuvB-like	0.003	High	8.995	11	1	6	1
H7C463	MICOS com	0	High	16.474	4	1	4	1
H0YN14	Importin-	0.01	High	3.33	3	1	1	1
P00441	Superoxic	0	High	62.067	40	3	93	3
P01718	Immunoglo	0.043	Medium	1.946	10	1	1	1
Q9Y2W1	Thyroid h	0	High	12.479	2	1	6	1
Q9HBR0	Putative	0.008	High	4.182	1	1	2	1
A0A0C4DH3	Immunoglo	0	High	12.634	25	2	2	2

A0A0B4J1U	Immunoglo	0.043	Medium	1.945	7	1	2	1
Q13449	Limbic sy	0.009	High	3.897	3	1	7	1
Q05639	Elongatio	0	High	30.03	10	2	81	1
P62841	40S ribos	0	High	24.826	19	1	12	1

# AAs	MW [kDa]	calc. pI	Score Sec	# Peptide	# Razor F	Found in	Found in	Found in
146	15.9	4.44	18.66	6	0	Peak Four	Peak Four	Peak Four
4061	434.9	9.25	5.11	3	0	Peak Four	Peak Four	Peak Four
381	42.6	5.59	92.9	2	0	High	High	High
318	36.1	5.81	1.72	1	0	Not Founc	Peak Four	Peak Four
1028	112.8	6.3	0	1	0	High	High	Peak Four
466	53.6	5.12	6.35	2	0	High	High	High
445	49.9	4.89	93.98	4	0	High	High	High
224	25	5.63	26.75	6	0	High	High	High
537	57.8	7.77	8.23	2	0	High	High	Peak Four
234	25.7	9.58	4.34	1	0	High	Peak Four	Not Founc
795	87.2	7.12	2.27	1	0	Not Founc	Not Founc	Peak Four
313	35.7	7.01	0	1	0	Not Founc	High	High
732	84.6	5.02	278.65	6	0	Peak Four	High	High
106	12.2	9.35	0	1	0	High	High	High
2570	275.3	6.49	0	1	0	High	Peak Four	Peak Four
270	30.6	6.38	254.55	5	0	Peak Four	High	Peak Four
332	36.7	8.27	50.34	6	0	High	High	High
759	87.6	7.78	0	1	0	High	High	High
466	51.6	7.23	42.7	4	0	High	High	High
1363	146.8	6.76	0	1	0	Peak Four	Peak Four	High
208	23.3	8.98	349.18	6	0	High	High	High
135	14.4	5.45	0	1	0	Peak Four	Peak Four	High
763	84.6	6.52	37.44	4	0	High	High	High
268	30.4	8.34	5.27	1	0	Peak Four	Peak Four	Peak Four
631	71.6	4.65	1.8	1	0	Peak Four	Peak Four	High
120	13.1	7.28	37.18	2	1	Not Founc	Peak Four	Not Founc
485	55.3	6.95	29.77	4	0	Peak Four	Peak Four	High
277	30.6	5.3	83.1	3	0	High	High	High
1018	113.2	5.9	23.55	7	0	High	Peak Four	High
117	12.8	8.28	17.94	1	0	High	High	High
1445	161.6	5.85	23.35	6	0	High	High	High
463	49.4	5.39	63.16	5	0	High	High	High
129	14	10.9	9.23	1	0	Peak Four	Peak Four	Peak Four
453	49.4	6.77	609.2	15	0	High	High	High
167	18.8	8.95	0	1	0	High	High	High
325	34.8	7.5	34.56	7	0	Peak Four	Peak Four	High
819	91.1	5.6	0	1	0	Peak Four	Not Founc	Peak Four
106	11.8	8.09	1.73	1	0	Not Founc	Not Founc	Peak Four
313	34.9	6.01	1.91	1	0	Not Founc	Not Founc	Not Founc
1121	129.5	6.4	0	1	0	Not Founc	Not Founc	Not Founc
406	46.2	6.99	2.98	1	0	Not Founc	Not Founc	High
543	62.6	6.8	2.27	1	0	Not Founc	Not Founc	Not Founc
681	78.8	4.86	3.18	1	0	Not Founc	Not Founc	Not Founc
870	99	4.94	0	1	0	Not Founc	Peak Four	Not Founc
1596	174.9	9.74	0	1	0	Not Founc	Not Founc	Not Founc
315	34.8	5.76	5.97	1	0	Not Founc	Not Founc	Not Founc
613	68.1	6.06	9.7	1	0	Not Founc	Not Founc	Not Founc
911	101.4	5.05	0	1	0	Not Founc	Not Founc	Not Founc
154	15.9	6.13	202.38	3	0	High	High	High
113	12.2	5.01	0	1	0	Not Founc	Not Founc	Not Founc
955	108.6	10.15	7.19	1	0	Not Founc	Not Founc	Not Founc
1119	119.7	5.73	2.32	1	0	Not Founc	Not Founc	Not Founc
117	12.7	8.27	5.54	2	0	Not Founc	Not Founc	Not Founc

121	13.5	9.2	0	1	0	Not Found	Not Found	Not Found
338	37.4	6.98	1.7	1	0	High	High	High
463	50.4	9.03	173.26	2	0	Not Found	Not Found	Not Found
145	17	10.39	22.27	1	0	Not Found	Not Found	Not Found

Found in	Found in	Found in	Found in	Found in	# Proteir	Modificat	Chenxq	Yuanxr
High	Not Founc	Not Founc	Not Founc	Not Founc	1		22.01555	21.33346
High	Not Founc	Peak Four	Not Founc	Not Founc	1		19.38639	19.25187
High	Peak Four	Peak Four	Not Founc	Not Founc	1		18.8256	23.10402
High	Not Founc	Not Founc	Not Founc	Not Founc	1		17.96292	19.04485
High	Not Founc	Not Founc	High	Not Founc	1		19.11572	18.81344
Peak Four	Not Founc	Not Founc	Not Founc	Not Founc	1		20.04671	20.27445
High	High	High	High	High	1		25.10577	23.05845
High	High	Peak Four	Peak Four	Peak Four	1		23.39992	23.08377
Peak Four	Peak Four	Peak Four	Not Founc	Peak Four	1		21.37348	20.13958
Not Founc	Not Founc	Not Founc	Not Founc	Not Founc	1		20.09437	18.90169
High	High	Not Founc	Not Founc	Not Founc	1		16.89481	17.64618
Peak Four	Not Founc	Not Founc	Not Founc	Not Founc	1		16.21974	18.48422
High	High	High	High	High	1		23.14979	23.28977
Peak Four	Peak Four	Peak Four	Peak Four	Peak Four	1		20.40004	19.40379
Peak Four	Peak Four	Not Founc	Peak Four	Peak Four	1		19.73829	19.62222
High	Peak Four	High	Peak Four	High	1		21.38847	21.95436
High	High	High	High	High	1		23.37046	23.69782
High	Peak Four	High	Peak Four	High	1		21.52097	21.45003
High	High	High	High	High	1		23.4425	23.1268
High	Peak Four	Peak Four	Not Founc	Peak Four	1		18.34344	18.49056
High	High	High	High	High	1		29.33014	29.81894
Peak Four	High	High	High	Peak Four	1		19.2646	18.53798
High	High	High	High	High	1		21.51145	21.22507
Peak Four	High	High	High	High	1		18.79607	18.59071
Peak Four	High	Peak Four	Peak Four	Peak Four	1		21.79099	22.10264
Not Founc	Not Founc	Peak Four	High	Peak Four	1		24.22417	24.25916
High	High	High	High	High	1		21.65412	21.30079
High	High	High	High	High	1		23.8515	24.00538
High	High	High	Peak Four	High	1		21.64304	21.36389
High	High	High	High	High	1		21.61789	21.26896
High	High	High	High	High	1		20.4327	20.8958
High	High	High	High	High	1		23.57375	24.55165
High	High	High	High	High	1		18.38503	18.38778
High	High	High	High	High	1		27.46437	28.36551
High	High	High	High	High	1		25.09098	23.93443
Peak Four	High	High	High	High	1		21.92111	22.06197
Not Founc	Peak Four	Peak Four	Not Founc	High	1		18.44606	15.71949
Not Founc	Peak Four	High	Peak Four	Peak Four	1		16.51997	15.72041
Not Founc	Peak Four	Not Founc	Not Founc	High	1		16.411	15.93908
Not Founc	Peak Four	High	Peak Four	High	1		16.03579	16.07765
Not Founc	Peak Four	Peak Four	Peak Four	Peak Four	1		15.33881	15.06622
Not Founc	Not Founc	High	Not Founc	High	1		16.32981	17.02827
Peak Four	High	Peak Four	Not Founc	Not Founc	1		14.70787	13.47443
Not Founc	Peak Four	High	High	High	1		14.89511	16.53554
Not Founc	Peak Four	Peak Four	Peak Four	High	1		17.55201	17.37582
High	High	High	Peak Four	High	1		16.7883	18.35122
Not Founc	High	High	Not Founc	Peak Four	1		15.75438	14.45299
Not Founc	Peak Four	Peak Four	High	Peak Four	1		15.1237	14.61857
High	High	High	High	High	1		20.38647	20.70401
Not Founc	Peak Four	Peak Four	High	Not Founc	1		15.0236	16.4648
Not Founc	High	High	High	High	1		16.03919	15.75938
High	Peak Four	Peak Four	Peak Four	High	1		16.43368	13.4489
Not Founc	Peak Four	Peak Four	High	Peak Four	1		14.87499	16.21022

Not Founc	High	Peak Four	High	Not Founc	1	15.30895	15.06907
High	High	Peak Four	High	High	1	17.02546	15.88842
Not Founc	High	High	High	High	1	16.08564	16.54519
Not Founc	High	High	High	High	1	15.13309	15.1595

Wangw	Zhanghd	Lianl	Wangy	Luoyh	Jinsh	Group_Cas	Group_Cor	Group_Cas
21.03839	24.24935	14.61002	17.2783	15.64995	18.60232	4683606	94967.61	49.31793
19.91949	22.1127	13.46884	19.7006	11.35839	16.91749	1177759	42094.03	27.97923
23.85696	21.04431	17.52489	18.98384	16.82887	14.46078	3425120	126571.7	27.06071
19.6715	20.27814	15.81665	15.8563	15.44311	14.58171	618900.9	43976.41	14.07348
18.16636	18.26614	16.24023	14.1598	15.68448	14.01645	394704.9	33346.39	11.83651
19.9749	17.49866	17.04698	15.23988	15.87497	16.07786	715542.8	68315.33	10.47412
21.91468	20.77558	20.69937	19.35162	18.8964	18.99322	6878296	733864.2	9.372709
22.80589	25.99662	21.93301	19.42064	21.09553	20.4687	14825197	1738568	8.527245
19.28298	17.78169	17.75032	17.16552	16.12869	16.76177	819527.3	126745.4	6.465936
18.54261	17.07892	17.43852	16.97679	14.42264	15.06188	412604.2	64408.23	6.406078
19.00107	19.19593	14.66676	16.44803	14.88129	16.57779	297906	51186.22	5.820043
18.88099	17.45861	16.37615	14.92342	14.01397	16.42144	222106.2	44260.91	5.018111
24.65839	24.18969	22.888	21.87978	21.61066	20.99214	14828917	3760896	3.942921
18.66815	18.13209	17.4511	17.34166	16.97886	16.96284	582143.3	148862.2	3.910618
19.05448	19.38454	18.51262	15.3203	18.01634	18.3071	716139.7	190454.8	3.760155
21.53034	22.49763	19.04674	20.53758	20.93223	20.03923	3761043	1154586	3.257481
23.92993	23.30043	22.36689	22.70182	22.0119	21.77569	12493386	4865215	2.5679
20.68253	21.95378	20.51457	20.51963	20.03228	20.73841	2770722	1433609	1.93269
23.90488	23.32006	23.01244	22.61479	22.23136	23.17852	11447742	7099460	1.612481
19.15925	19.33833	18.43997	17.95875	17.93889	18.24515	466944.6	289999.8	1.610155
29.24723	29.49916	28.59294	29.08454	28.87366	28.68636	7.46E+08	4.7E+08	1.585009
19.29459	18.8138	19.85038	19.57611	19.6504	19.1995	516261.9	777830.9	0.66372
21.85792	21.41354	22.24034	22.15435	21.82237	22.16853	2969924	4484129	0.662319
19.42457	18.21902	19.5633	19.66887	19.0615	19.61341	443198.4	729612.2	0.607444
22.05436	22.39298	23.06119	22.17713	22.73043	23.31953	4449594	7415272	0.600058
24.57181	23.90149	24.69303	24.61901	25.62653	24.99094	19802237	33147092	0.597405
21.5221	22.0186	22.54631	21.89678	22.36727	22.88957	3231790	5631098	0.573918
23.7264	24.28841	24.67763	25.37316	24.42963	24.80836	16408325	29663677	0.553145
21.831	21.01418	22.43494	22.70984	21.78386	22.4156	2890779	5294501	0.545997
21.94371	20.89095	22.02451	22.58711	23.03689	21.74002	2826091	5335284	0.529698
21.25579	20.33842	21.58175	21.87304	21.57918	21.60581	1740029	3313547	0.525126
24.45059	23.37601	24.4564	25.46934	25.17821	25.19718	16638234	35351831	0.470647
18.67254	19.13945	19.89746	19.68733	19.92307	19.66395	410265.6	908390.1	0.45164
27.8138	27.23074	29.08183	28.09222	30.14815	28.90542	2.21E+08	5.58E+08	0.395486
25.02481	23.7147	26.33949	25.3725	25.84517	25.7063	22779454	59067754	0.38565
23.21657	20.64719	23.88894	23.54561	24.5895	23.27919	4084456	14869063	0.274695
18.17655	16.89058	19.90792	19.69324	18.68598	19.34689	162285	695894.1	0.233204
18.91558	15.59114	18.14645	19.37266	18.78983	18.84347	105491.9	452671.7	0.233043
16.23585	16.17723	18.85071	20.12356	16.03717	18.26253	74801.96	326900.6	0.228822
16.32585	15.03765	18.78326	19.53291	17.72399	17.48946	59856.94	341708.4	0.17517
18.72743	14.23462	18.01646	18.46446	18.49404	18.48157	58728.43	337407.4	0.174058
15.57333	15.50432	19.04089	19.18942	17.52413	19.27833	70676.09	443382.4	0.159402
16.30276	17.88125	18.91281	18.22118	17.8063	18.22635	49377.78	320877.4	0.153884
15.01769	15.78956	18.59563	18.84509	18.62052	17.87733	48291.32	366801.9	0.131655
18.27104	15.36223	19.33471	20.62961	20.6516	19.72065	144456.5	1111550	0.12996
16.96625	15.82101	20.82775	20.70262	19.62107	18.59549	129419.6	1003585	0.128957
16.87697	15.012	20.6327	19.08471	16.95759	17.70038	47121.07	395644.3	0.1191
15.6156	15.66941	17.73524	18.8185	19.20378	17.57829	39152.55	330422.5	0.118492
21.86151	21.80166	24.99497	25.25208	24.16524	22.71859	2389725	20409281	0.11709
16.78779	14.34036	20.209	19.50113	20.75477	15.54049	51566.19	524778.5	0.098263
16.17366	15.15651	20.05258	20.34294	20.15146	16.0417	56352.25	580594.2	0.09706
15.77601	19.70798	19.72224	19.43868	19.77851	19.909	83047.06	858886.3	0.096692
16.06604	15.57977	19.27021	19.55033	20.46167	17.76975	52599.36	629126.6	0.083607

16.85746	15.0195	19.10161	18.93622	21.00002	17.57915	48434.36	583450.3	0.083014
20.17558	14.56389	20.78135	20.65499	20.08789	20.91956	123430.6	1601449	0.077074
15.59016	14.27938	20.51656	21.34543	20.82426	20.03419	50538.4	1680091	0.030081
14.93041	16.20344	21.92036	22.657	22.64522	22.17356	41956.56	5342319	0.007854

pvalue
0.002641
0.049734
0.017845
0.000572
0.000995
0.004097
0.018914
0.014262
0.017439
0.031318
0.014475
0.031825
0.010135
0.008154
0.045987
0.011814
0.001613
0.020265
0.04272
0.045336
0.007237
0.041184
0.010518
0.04706
0.036857
0.031886
0.020238
0.010615
0.016406
0.046922
0.006127
0.025958
0.000943
0.034002
0.015586
0.02054
0.021841
0.041096
0.048081
0.003957
0.044669
0.002901
0.033679
0.000526
0.005874
0.007095
0.019021
0.000604
0.004001
0.044034
0.019295
0.040595
0.001332

0.004882
0.022482
0.000105
8.55E-07
