# Supplementary material for: Role of increased IGFBP2 in trophoblast cell proliferation and recurrent spontaneous abortion development: A pilot study
Source: Physiol Rep. 2024 Feb 5;12(3):e15939. doi: 10.14814/phy2.15939 (PMC10843903; doi:10.14814/phy2.15939)
Supplement: Supplementary file 2 — Tables S1–S3. [file PHY2-12-e15939-s001.zip › Captions.docx]

Table S1: All identified proteins in plasma samples collected from pregnant women with (Case) and without (Control) recurrent spontaneous abortion.

Table S2: Differentially expressed proteins in plasma samples collected from pregnant women with (Case) vs. without (Control) recurrent spontaneous abortion.

Table S3: Detailed information including Gene Ontology (GO) and Kyoto Encyclopedia of Genes and Genomes (KEGG) of differentially expressed genes in trophoblast cells treated with (IGFBP2) or without (Control) IGFBP2.
